# Hepatic Suppression of Mitochondrial Complex II Assembly Drives Systemic Metabolic Benefits

**DOI:** 10.1002/advs.202105587

**Published:** 2022-01-17

**Authors:** Xueqiang Wang, Weiqiang Lv, Jie Xu, Adi Zheng, Mengqi Zeng, Ke Cao, Xun Wang, Yuting Cui, Hao Li, Meng Yang, Yongping Shao, Fang Zhang, Xuan Zou, Jiangang Long, Zhihui Feng, Jiankang Liu

**Affiliations:** ^1^ Center for Mitochondrial Biology and Medicine The Key Laboratory of Biomedical Information Engineering of Ministry of Education School of Life Science and Technology Xi'an Jiaotong University Xi'an Shaanxi 710049 China; ^2^ Frontier Institute of Science and Technology Xi'an Jiaotong University Xi'an Shaanxi 710049 China; ^3^ Department of Ophthalmology Shanghai General Hospital Shanghai Jiao Tong University School of Medicine Xi'an Shanghai 200240 China; ^4^ National Clinical Research Center for Eye Diseases Shanghai 200240 China; ^5^ National & Local Joint Engineering Research Center of Biodiagnosis and Biotherapy The Second Affiliated Hospital of Xi'an Jiaotong University Xi'an Shannxi 710004 China; ^6^ Shaanxi Provincial Clinical Research Center for Hepatic & Splenic Diseases The Second Affiliated Hospital of Xi'an Jiaotong University Xi'an Shannxi 710004 China; ^7^ University of Health and Rehabilitation Sciences Qingdao Shandong 266071 China

**Keywords:** complex II, dietary restriction, insulin sensitivity, mitochondria, SDHAF4

## Abstract

Alternate day fasting (ADF), the most popular form of caloric restriction, has shown to improve metabolic health in preclinical subjects, while intrinsic network underpinning the process remains unclear. Here, it is found that liver undergoes dramatic metabolic reprogramming during ADF, accompanied surprisingly with unique complex II dysfunction attributing to suspended complex II assembly via suppressing SDHAF4, a recently identified assembly factor. Despite moderate mitochondrial complex II dysfunction, hepatic *Sdhaf4* knockout mice present intriguingly improved glucose tolerance and systemic insulin sensitivity, consistent with mice after ADF intervention. Mechanistically, it is found that hepatocytes activate arginine‐nitric oxide (NO) biosynthesis axle in response to complex II and citric acid cycle dysfunction, the release of NO from liver can target muscle and adipocytes in addition to its autocrine action for enhanced insulin sensitivity. These results highlight the pivotal role of liver in ADF‐associated systemic benefits, and suggest that targeting hepatic complex II assembly can be an intriguing strategy against metabolic disorders.

## Graphical abstract

Alternate day fasting (ADF) decreases complex II activity in liver via suppressing its assembly. Such suppression drives compensatory activation of arginine metabolism, resulting in hepatic release of nitric oxide for promoting systemic insulin sensitivity. The study sets an unique example supporting diverse effects of mitochondrial function in maintaining health, and raising interests in further exploration of intricate mitochondrial metabolic network.

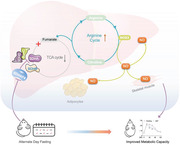

## 1 Introduction

Metabolic remodeling, either as an intervention of caloric restriction (CR), diet composition, or simply fasting, has been shown to improve metabolic syndrome‐associated declines in most pathophysiological parameters and to extend mean lifespan in various animal species.^[^ ^1^, ^2^, ^3^, ^4^, ^5^, ^6^ ^]^ The underlying physiological processes involve periodic shifts of metabolic fuel sources, promoting repair mechanisms of tissues, and optimizing energy utilization for cellular and organismal health,^[^ ^3^, ^7^, ^8^ ^]^ while the detail mechanisms remain elusive.

Alternate day fasting (ADF), as a means of dietary restriction gaining popularity, consists of a day of ad libitum eating followed by a day with no caloric consumption.^[^ ^9^ ^]^ Recent studies have achieved intriguing progress revealing the benefits of ADF in both the animal models and the clinical subjects. ADF was shown to significantly promote weight loss, glucose tolerance, and insulin sensitivity in diabetic mice.^[^ ^10^, ^11^ ^]^ Clinical benefits in obese adults such as weight loss and cardio‐protection were also reported.^[^ ^12^ ^]^ More importantly, Stekovic and colleagues^[^ ^13^, ^14^ ^]^ reported that ADF could improve physiological and molecular markers of aging in healthy, nonobese humans, suggesting ADF as a short‐term safe and effective intervention for weight management and metabolic disease risk reduction. These studies suggest that ADF may effectively modulate several risk factors with insulin signaling being the primary one, thereby preventing chronic diseases.^[^ ^9^ ^]^ Interestingly, human trials to date have reported greater insulin mediated glucose uptake without affecting fasting glucose or insulin concentrations in healthy subjects,^[^ ^9^ ^]^ indicating a primary regulation on insulin sensitivity by ADF, while the driving mechanisms remain largely unknown.

In the aspect of insulin action and glucose management, liver has been well accepted for playing vital role under either caloric restriction or feeding,^[^ ^15^, ^16^, ^17^ ^]^ which is inextricably interwound with complex mitochondrial metabolism including the tricarboxylic acid (TCA) cycle, *β* oxidation, glutamic acid, and aspartic acid metabolism.^[^ ^18^, ^19^, ^20^, ^21^ ^]^ Yet, whether and how hepatic mitochondria are involved in ADF‐mediated metabolic benefits is unexplored. Here, we report that ADF promotes hepatic metabolic reprogramming with uniquely suppressed mitochondrial complex II activity due to disrupted assembly. We demonstrate that succinate dehydrogenase (SDH) assembly factor 4 (SDHAF4), the newly identified factor for complex II assembly, was decreased in the liver under ADF intervention leading to complex II assembly dysfunction. Intriguingly, such suppression of complex II derived mitochondria fighting back for compensatory activation of arginine/nitric oxide cycle, resulting in hepatic release of NO for promoting systemic insulin sensitivity. The present study reveals a novel network accounting for ADF‐mediated metabolic benefits, shedding light on future clinical treatment of metabolic disorders. Moreover, the study sets a unique example supporting diverse effects of mitochondrial function in maintaining health, raising interests in further exploration of intricate mitochondrial metabolic network.

## 2 Results

### 2.1 ADF Promotes Hepatic Metabolic Remodeling with Disrupted Complex II Assembly

**Figure 1:**
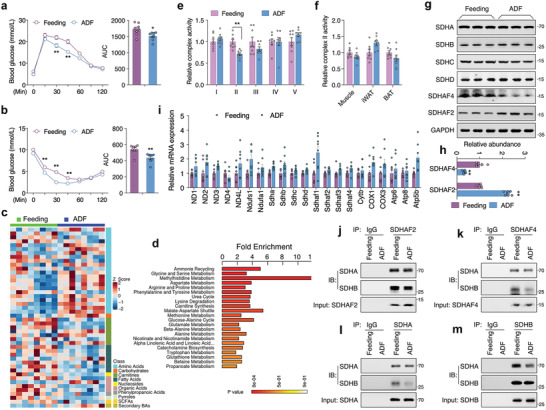
ADF promotes hepatic metabolic remodeling with disrupted complex II assembly. a) Glucose tolerance test and b) insulin tolerance test in mice under regular feeding or ADF intervention for 4 weeks, *n* = 8. c) Metabolomics analysis for liver of mice under regular feeding or ADF intervention for 4 weeks, with *Z*‐score plot of global metabolic profiles among all the samples and d) metabolic pathway enrichment analysis, *n* = 8. e) Mitochondrial electron transport chain complex activities in liver, *n* = 8. f) Complex II activity in skeletal muscle, inguinal white adipose tissue (iWAT), and brown adipose tissue (BAT), *n* = 8. g) Immunoblots analysis for complex II subunits and assembly factors SDHAF2 and SDHAF4 in liver, representative blotting images, h) summary analysis of arbitrary unit. i) mRNA levels of representative mitochondrial complex subunits in liver, *n* = 8. j–m) Coimmunoprecipitation of j) SDHAF2 with SDHA/SDHB, k) SDHAF4 with SDHA/SDHB, l) SDHA with SDHB, and m) SDHB with SDHA from the lysates of mice liver after ADF intervention. Values are mean ± SEM, *p < 0.05, **p < 0.01.

Initial ADF was referred to the pattern of a “feast day” followed by a “fast day,”^[^ ^6^ ^]^ which was carried out in the present study. Mice were under regular feeding or ADF intervention for 4 weeks with body weight and food intake monitored every 4 days. Mice under ADF showed lower body weight gain starting 1 week after intervention (Figure S1a, Supporting Information), while food intake was comparable between two groups expect slight decrease in ADF group at first week (Figure S1b, Supporting Information). Meanwhile, blood analysis revealed that ADF had no significant effects on serum ALT, AST, TC, TG, creatinine, urea, and fasting insulin levels in mice (Figure S1c–i, Supporting Information), while glucose tolerance and insulin sensitivity were moderately but significantly improved by ADF (**Figure** **1**a,b), consisting with previous observations on obese and diabetic mice.^[^ ^22^, ^23^ ^]^ As liver being the pivotal tissue for metabolic regulation, metabolomics analysis of liver tissues identified 214 detectable metabolites, and among which 47 compounds showed significant alterations between ADF and regular feeding group (Figure 1c). Further enrichment analysis revealed the changes were primarily associated with amino acid metabolism (Figure 1d and Figure S1j, Supporting Information).

Mitochondria, the key organelle coupling nonessential amino acid metabolism with TCA cycle, were thereby investigated. Surprisingly, among the five complexes on electron transport chain (ETC), the activity of complex II was specifically decreased in ADF group (Figure 1e), while not affected in peripheral tissues including skeletal muscle, inguinal white adipose tissue (iWAT), and brown adipose tissue (BAT) (Figure 1f). Complex II or SDH^[^ ^24^ ^]^ is comprised of four subunits, mRNA and protein levels of which were unaffected in ADF group (Figure 1g,h). In addition, mRNA levels of other mitochondrial complex subunits were also unaltered by ADF (Figure 1i). Instead, the protein levels of SDHAF2 and SDHAF4, two assembly factors initiating the assembly of SDH complex by promoting SDHA maturation and binding with SDHB,^[^ ^25^, ^26^ ^]^ were significantly altered by ADF (Figure 1g,h). Even though SDHAF2 protein was slightly increased, the binding activity of SDHAF2 with SDHA and SDHB was not affected (Figure 1j). Instead, protein level of SDHAF4 was dramatically decreased by ADF (Figure 1g,h), and the binding of SDHAF4 with SDHB was decreased (Figure 1k), thereby disrupted further binding of SDHA with SDHB (Figure 1l,m). Thus, the decreased complex II activity observed after ADF intervention was primarily attributed to suspended SDH assembly due to downregulated SDHAF4.

### 2.2 Hepatic Deficiency of *Sdhaf4* in Mice Maintains Normal Phenotype with Disrupted SDH Complex

**Figure 2:**
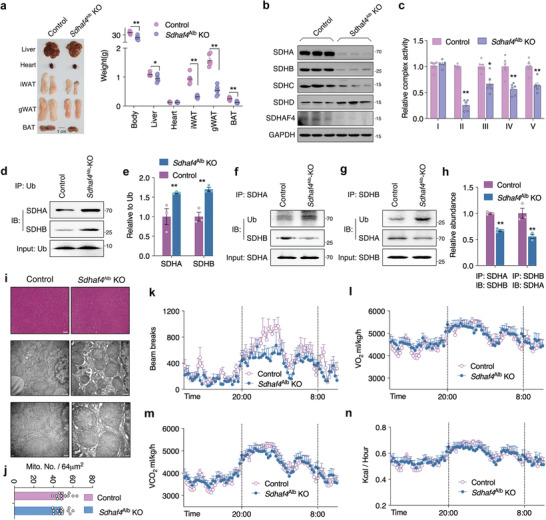
Hepatic *Sdhaf4* knockout mice maintain normal phenotype with disrupted SDH complex. The control and *Sdhaf4* ^Alb^ KO mice were analyzed at the age of 16 weeks: a) Representative images of tissues and summary analysis of body and tissue weight, *n* = 8. b) Immunoblots for complex II subunits in liver of control and *Sdhaf4* ^Alb^ KO mice, representative blotting images, c) summary analysis of arbitrary unit, *n* = 8. d,e) Immunoprecipitation of ubiquitin for blot analysis of SDHA and SDHB from liver lysates of control and *Sdhaf4* ^Alb^ KO mice, *n* = 3. f–h) Co‐immunoprecipitation of SDHA with SDHB, SDHB with SDHA, for analysis of SDHA/SDHB binding from liver lysates of control and *Sdhaf4* ^Alb^ KO mice, *n* = 3. i,j) HE staining and transmission electron microscope (TEM) analysis of liver section in control and *Sdhaf4* ^Alb^ KO mice, i) representative images, j) analysis of mitochondrial per area. k) Metabolic cage analysis of control and *Sdhaf4* ^Alb^ KO mice, locomotor activity, l) whole body oxygen consumption rate, m) CO_2_ production rate, and n) heat generation, *n* = 8. Values are mean ± SEM, *p < 0.05, **p < 0.01.

To explore the detail involvement of hepatic SDHAF4‐mediated complex II dysfunction in ADF metabolic benefits, we generated mice with liver‐specific deletion of the *Sdhaf4* gene using albumin‐cre (*Sdhaf4* ^Alb^‐KO: *Sdhaf4^f/f^* × albumin‐cre vs control: *Sdhaf4^f/f^*). The KO mice presented regular feeding and growing phenotype, except for a suppressed body weight gain in both male and female KO mice (Figure S2a–d, Supporting Information), and the decreased body weight was primary in liver and fat tissues at the age of 16 weeks (**Figure** **2**a). Blood analysis revealed that hepatic *Sdhaf4* knockout had no significant effects on serum ALT, AST, TC, TG, and creatinine levels in mice expect for a slight increase in urea (Figure S2e–j, Supporting Information). Consistent with ADF mice, *Sdhaf4* ^Alb^‐KO presented comparable expression levels of mitochondrial complex subunits with control mice (Figure S2k, Supporting Information), while protein levels of SDH subunits SDHA and SDHB showed profound decrease (Figure 2b), accompanied by a dramatic decrease of complex II activity, as well as moderate decrease of complex III, IV, and V activities in *Sdhaf4* ^Alb^‐KO mice (Figure 2c). IP analysis of SDHA/SDHB binding and ubiquitin modification (Figure 2d–h) indicated that the decrease of SDH protein levels was attributed to enhanced ubiquitin modification and protein degradation as suggested by Van Vranken et al. in previous report.^[^ ^26^ ^]^ As expected, such decrease of complex II activity would negatively affect mitochondrial adenosine triphosphate (ATP) production, which was found moderately decreased in both *Sdhaf4* ^Alb^‐KO mice and mice under ADF intervention (Figure S3a,b, Supporting Information). However, the lowered energy production did not alter AMPK and mTOR signaling, as well as mitophagy activity (Figure S3c, Supporting Information). Consistently, hematoxylin and eosin (H&E) staining showed normal hepatic structure in the *Sdhaf4* ^Alb^‐KO mice (Figure 2i). Electron microscopy revealed vacuolous structure rich in *golgi* bodies distributed around mitochondria in the KO mice, while mitochondrial microstructure and number was not altered in the KO mice (Figure 2i,j). All above results suggest that hepatic ablation of SDHAF4 moderately suppress mitochondrial activity without provoking dramatic mitochondrial stress.

Surprisingly, the *Sdhaf4* ^Alb^‐KO mice presented normal energy metabolism including total activity, O_2_ consumption, CO_2_ production, heat generation, food intake, and respiratory exchange (Figure 2k–n and Figure S3d,e, Supporting Information), expect for a decrease of ambulatory activity (Figure S3f, Supporting Information). To further clarify the energy absorption/utilization in the *Sdhaf4* ^Alb^‐KO mice, we first analyzed the feces energy of both WT and *Sdhaf4* ^Alb^‐KO mice, and found comparable changes on both feces composition and calories (Figure S3g,h, Supporting Information). Further analysis of bile acid profile in the liver identified total 44 primary and secondary bile acids, while only 2 metabolites were found moderately changed (Figure S3i–m, Supporting Information), including chenodeoxycholic acid (CDCA) and 7‐keto lithocholic acid (7‐Keto LCA), which is a secondary bile acid and usually presents extremely low abundance in liver. Collectively, we speculate that hepatic deficiency of SDHAF4 did not raise significant impact on energy absorption/utilization in mice.

### 2.3 Hepatic *Sdhaf4* Knockout Improves Systemic Metabolic Sensitivity in Mice

**Figure 3:**
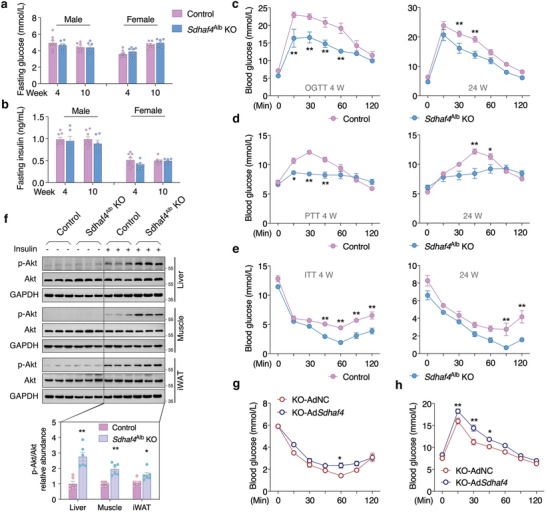
Hepatic *Sdhaf4* knockout improves systemic metabolic sensitivity in mice. a) Fasting glucose and b) fasting insulin in both male and female mice at the age of 4 and 10 weeks, *n* = 8 for control mice, *n* = 6 for *Sdhaf4* ^Alb^ KO mice. c) Glucose tolerance test, d) pyruvate tolerance test, and e) insulin tolerance test in male control and *Sdhaf4* ^Al b^ KO mice at the age of 4 and 24 weeks separately. f) Immunoblots analysis of p‐Akt level in iWAT, muscle, and liver tissues of control and *Sdhaf4* ^Alb^ KO mice with or without insulin challenge, *n* = 6. g,h) Glucose tolerance test and insulin tolerance test in *Sdhaf4* ^Alb^ KO mice after adenovirus‐mediated hepatic *Sdhaf4* overexpression for 2 weeks, *n* = 6. Values are mean ± SEM, *p < 0.05, **p < 0.01.

In addition to normal serum lipids between *Sdhaf4* ^Alb^‐KO and control mice, we also found comparable fasting glucose and fasting insulin levels between them (**Figure** **3**a,b). Intriguingly, consistent with ADF intervention, *Sdhaf4* ^Alb^‐KO mice presented sustained improvement on glucose tolerance and insulin sensitivity evidenced by the tests of oral glucose tolerance, pyruvate tolerance, and insulin tolerance in mice at age of 4 and 24 weeks (Figure 3c–e). As expected, *Sdhaf4* ^Alb^‐KO mice showed significant increased Akt phosphorylation in liver, muscle (quadriceps femoris), and inguinal white adipose tissue (iWAT) after in vivo insulin challenge (Figure 3f). To further verify that loss of hepatic *Sdhaf4* was the direct cause for metabolic benefits in mice, an adenovirus‐based infection was employed for the transient expression *Sdhaf4* in liver (Figure S4a, Supporting Information). Overexpression of *Sdhaf4* significantly restored SDHA and SDHB protein levels in the liver of *Sdhaf4* ^Alb^‐KO mice (Figure S4b, Supporting Information), without affecting serum lipids in either control or *Sdhaf4* ^Alb^‐KO mice (Figure S4c–f, Supporting Information). Meanwhile, hepatic overexpression of *Sdhaf4* did not alter glucose and insulin tolerance in control mice (Figure S4g,h, Supporting Information), but dramatically decreased the metabolic capability in *Sdhaf4* ^Alb^‐KO mice (Figure 3g,h). As expected, insulin challenged Akt phosphorylation was also decreased in the KO mice after restoring SDHAF4 level (Figure S3i, Supporting Information), further supporting the driving effect of hepatic SDHAF4 loss in improving systemic metabolic capacity. More importantly, aging observation of *Sdhaf4* ^Alb^‐KO mice up to 12 months showed comparable phenotype with control mice (Figure S5a, Supporting Information), grip strength test for mice limb strength (Figure S5b, Supporting Information) and open filed test for mice emotional and motor activity (Figure S5c–f, Supporting Information) also revealed comparable alterations between control and *Sdhaf4* ^Alb^‐KO mice at age of 2, 6, and 12 months, these observations suggest that suppression of hepatic complex II assembly could be safe and interesting strategy for improving systemic metabolic benefits.

**Figure 4:**
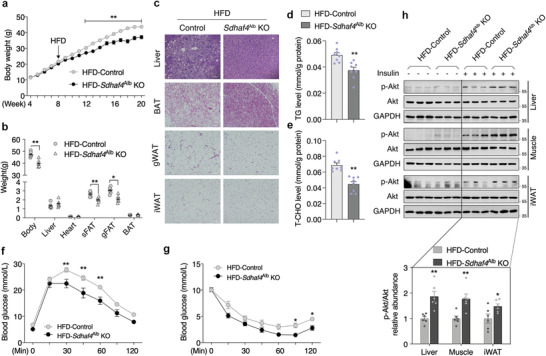
Loss of SDHAF4 in liver protects mice against metabolic stress. a) Body weight curve of control and *Sdhaf4* ^Alb^ KO mice under high fat diet (HFD) from age of 8 to 20 weeks, *n* = 6. b) Body and tissue weights of control and *Sdhaf4* ^Alb^ KO mice under HFD for 12 weeks, *n* = 6. c) HE staining for liver, BAT, gWAT, and iWAT sections of control and *Sdhaf4* ^Alb^ KO mice under HFD. d) Analysis of TG level and e) T‐CHO level in the liver of control and *Sdhaf4* ^Alb^ KO mice under HFD. f) Glucose tolerance test, g) insulin tolerance test of male control and *Sdhaf4* ^Alb^ KO mice after HFD for 12 weeks, *n* = 6. h) Immunoblots analysis of p‐Akt level in iWAT, muscle, and liver tissues of control and *Sdhaf4* ^Alb^ KO mice under HFD with or without insulin challenge, *n* = 3. Values are mean ± SEM, *p < 0.05, **p < 0.01. TG, triglycerides; T‐CHO, total cholesterol; gWAT, gonadal white adipose tissue.

Since hepatic SDHAF4 ablation presented improved metabolic capacity, we further evaluated the benefits against metabolic stress. After feeding on high fat diet (HFD) for 12 weeks, *Sdhaf4* ^Alb^‐KO mice showed significant resistance to HFD‐induced body weight gain (**Figure** **4**a), as well as increase of WAT (Figure 4b), which were supported by H&E staining of the liver, brown adipose tissue (BAT), gonadal WAT (gWAT), and iWAT (Figure 4c). In addition, biochemical analysis of liver triglycerides and total cholesterol level indicated significant decreased lipid accumulation in the liver of *Sdhaf4* ^Alb^‐KO mice under HFD challenge (Figure 4d,e), which was consistent with H&E staining of liver section. In line with normal diet feeding, *Sdhaf4* ^Alb^‐KO still showed significant improved glucose and insulin tolerance under HFD feeding (Figure 4f,g). As expected, a significant increase in insulin‐stimulated Akt phosphorylation in liver, muscle, and iWAT of *Sdhaf4* ^Alb^‐KO mice under HFD was also observed (Figure 4h). Collectively, above data further supported a sustained metabolic improvement in *Sdhaf4* ^Alb^‐KO mice under either regular or HFD environment.

### 2.4 Suppressed Complex II Assembly Mobilizes Amino Acid Metabolism

**Figure 5:**
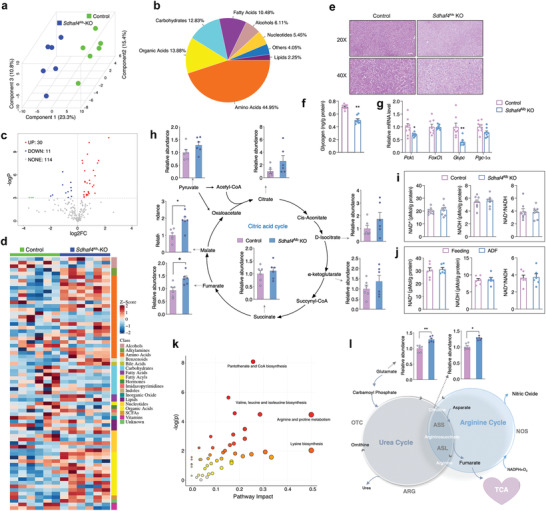
Suppressed complex II assembly mobilizes amino acid metabolism. a) PLS‐DA scores plot revealing classifications of the samples, b) metabolite classes and compositions, c) enhanced volcano plot showing the differential metabolites, d) *Z*‐score plot of global metabolic profiles among all the samples, *n* = 6. e) PAS staining of liver sections and f) biochemical analysis of liver tissues from control and *Sdhaf4* ^Alb^ KO mice for evaluating glycogen level, *n* = 8. g) mRNA levels of *Pck1*, *FoxO1*, *G6pc*, *Pgc‐1α* in livers of control and *Sdhaf4* ^Alb^ KO mice, *n* = 8. h) Biochemical analysis of TCA metabolites in liver of control and *Sdhaf4* ^Alb^ KO mice, *n* = 6. i) Level of NAD^+^, NADH, and NAD^+^/NADH in the liver of control and *Sdhaf4* ^Alb^ KO mice, *n* = 8. j) Level of NAD^+^, NADH, and NAD+/NADH in the liver of mice under regular feeding or ADF intervention for 4 weeks, *n* = 6. k) Metabolic pathway enrichment analysis comparing the −log(*p*) to the impact on the various pathways for the network metabolites, *n* = 6. l) Representative image of urea, arginine biosynthesis, and TCA cycle. Values are mean ± SEM, *p < 0.05, **p < 0.01.

Among the five mitochondrial respiratory complexes, complex II is the only known complex that participating in both TCA cycle and the electron transport chain. Loss of SDHAF4 disrupted complex II assembly thereby promoting SDH subunits degradation as indicated above. Thereby, we assumed a dramatic suppression of TCA activity would occur in the liver of *Sdhaf4* ^Alb^‐KO mice. Interestingly, metabolomics analysis of liver tissues in both control and *Sdhaf4* ^Alb^‐KO mice identified 155 metabolites, and only 41 of them were altered significantly (**Figure** **5**a–d), which presented more amino acids and less carbohydrates accumulated in the liver of *Sdhaf4* ^Alb^‐KO mice (Figure 5d). Biochemical assay and periodic acid‐Schiff (PAS) staining of liver section consistently presented decreased glycogen level in *Sdhaf4* ^Alb^‐KO mice (Figure 5e,f). Quantitative polymerase chain reaction (qPCR) analysis showed significant decreased glucogenic genes *Pgc‐1*, *FoxO1*, *Pck1*, and *G6pc* (Figure 5g) indicating suppressed gluconeogenesis activity, which may be attributed to increased insulin signaling as previously reported.^[^ ^27^ ^]^ More importantly, hepatic loss of SDHAF4 only affected fumarate and malate level among TCA metabolites, and instead of decrease, their levels were surprisingly increased in *Sdhaf4* ^Alb^‐KO mice (Figure 5h), suggesting an existence of compensatory pathways in response to SDH dysfunction. Even though, ATP level was moderately decreased in both *Sdhaf4* ^Alb^‐KO mice and mice under ADF intervention (Figure S3a,b, Supporting Information), the NAD^+^ and NADH level was not affected in either mice model (Figure 5i,j), indicating the KO mice and ADF intervened mice enabled a functional TCA cycle despite suppressed SDH activity. Further pathway enrichment analysis showed that amino acid metabolism pathways were the most affected ones (Figure 5k). Arginine metabolism pathway was suggested to be closely linked with TCA cycle in previous reports.^[^ ^28^, ^29^ ^]^ As shown in Figure 5g, arginine is regenerated by arginosuccinate synthase (ASS) and arginosuccinate lyase (ASL) with fumarate as byproduct, while malate, the product of fumarase action on fumarate, is converted to oxaloacetate for production of aspartate to join arginine biosynthesis cycle. Meanwhile, arginase (ARG) catalyzes the cleavage of arginine to ornithine and urea to proceed the urea cycle. We thus propose that suppressed complex II assembly activates arginine biosynthesis pathway to sustain fumarate level for maintaining TCA metabolic function in vivo.

### 2.5 Hepatic Loss of SDHAF4 Activates Arginine‐NO Cycle to Improve Insulin Sensitivity in Mice

**Figure 6:**
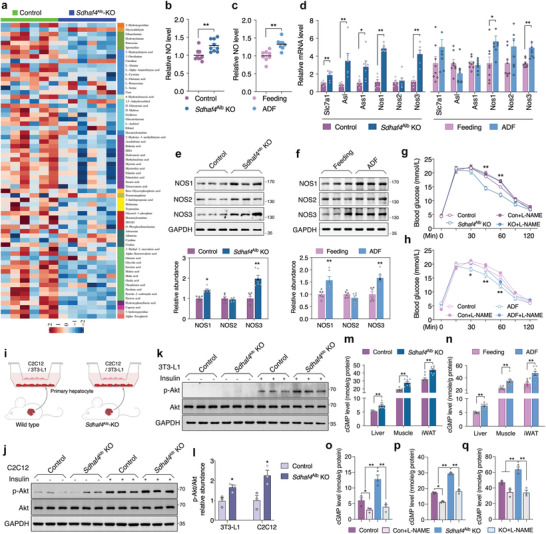
Hepatic loss of SDHAF4 activates arginine‐NO cycle to improve insulin sensitivity in mice. a) Metabolomics analysis for *Z*‐score plot of global metabolic profiles among all the serum samples of control and *Sdhaf4* ^Alb^ KO mice, *n* = 6. b) NO level in serums of control and *Sdhaf4* ^Alb^ KO mice, *n* = 8. c) NO level in serums of regular feeding and ADF intervened mice, *n* = 8. d) mRNA levels of *Slc7a1*, *Asl*, *Ass1*, *Nos1*, *Nos2*, and *Nos3* in livers of control and *Sdhaf4* ^Alb^ KO mice or regular feeding and ADF intervened mice, *n* = 6. e,f) Immunoblots of NOS1, NOS2, and NOS3 in livers of control and *Sdhaf4* ^Alb^ KO mice or regular feeding and ADF intervened mice, *n* = 6. g) Glucose tolerance test in control and *Sdhaf4* ^Alb^ KO mice with or without L‐NAME treatment for 2 weeks, *n* = 6. h) Glucose tolerance test in regular feeding and ADF intervened mice with or without L‐NAME treatment for 4 weeks, *n* = 6. i) Graphic illustration of cells co‐culture experiments. j) Immunoblots of p‐Akt in C2C12 cells and k) 3T3‐L1 cells that co‐cultured with primary hepatocytes from control and *Sdhaf4* ^Alb^ KO mice followed with or without insulin challenge for 15 min, l) statistical analysis of p‐Akt level, *n* = 3. m) cGMP level in the liver, muscle, and iWAT from control and *Sdhaf4* ^Alb^ KO mice at the age of 8 weeks, *n* = 8. n) cGMP level in the liver, muscle, and iWAT from regular feeding and ADF intervened mice for 8 weeks, *n* = 8. o) cGMP level in the liver, p) muscle, and q) iWAT from control and *Sdhaf4* ^Alb^ KO mice with or without L‐NAME treatment for 2 weeks, *n* = 3. Values are mean ± SEM, *p < 0.05, **p < 0.01.

Hepatic loss of SDHAF4 showing systemic improvement of insulin sensitivity indicates the existence of circulating mediator release from liver. To identify the potential effector, metabolomics analysis through liquid chromatography–mass spectrometry (LC‐MS) was used to profile comprehensive metabolites in the serum of control and *Sdhaf4* ^Alb^‐KO mice (Figure S6a–c. Supporting Information and **Figure** **6**a). Interestingly, enrichment analysis suggested arginine biosynthesis being top one affected pathway in *Sdhaf4* ^Alb^‐KO mice (Figure S6d, Supporting Information) with consistently increased citrulline (Figure 6a). Since production of citrulline is catalyzed by nitric oxide synthases (NOS) with NO, a crucial regulator in diverse physiological processes, the key byproduct (Figure 5g),^[^ ^30^ ^]^ we thereby wonder whether NO is the key effector mediating the metabolic benefits. Further experiments revealed enhanced NO level in the serum of both *Sdhaf4* ^Alb^‐KO and ADF intervened mice comparing to respective controls (Figure 6b,c). In addition, consistent increase of NOS1 and NOS3 expression was also observed in both *Sdhaf4* ^Alb^‐KO and ADF intervened mice (Figure 6d–f), while such increase was not observed in other tissues including BAT, iWAT, and muscle (Figure S7a–f, Supporting Information), suggesting an arginine‐NO cycle is activated in the liver of both *Sdhaf4* ^Alb^‐KO and ADF intervention. Intriguingly, administration of NOS inhibitor N(G)‐nitro‐L‐arginine methyl ester (L‐NAME) through drinking water to inhibit NO production in *Sdhaf4* ^Alb^‐KO and ADF intervened mice (Figure S8a,b, Supporting Information) could dramatically eliminate their metabolic advantage on glucose tolerance (h) and insulin‐associated Akt phosphorylation (Figure S8c–g, Supporting Information). Moreover, administration of L‐NAME abolished the benefits of *Sdhaf4* ^Alb^‐KO mice against HFD‐induced metabolic stress (Figure S6h–j, Supporting Information). To directly illustrate the metabolic regulatory effects of liver on muscle and adipose tissues, a co‐culture of primary hepatocytes with mouse C2C12 myotubes or 3T3‐L1 adipocytes was performed (Figure 6i). Data showed significant increase of Akt phosphorylation in C2C12 or 3T3‐L1 cells that co‐cultured with hepatocytes from *Sdhaf4* ^Alb^‐KO mice (Figure 6j–l). To demonstrate that circulating NO acted on target tissues, the known NO downstream messenger cGMP was analyzed. Data showed significantly increased cGMP level was observed in the liver, muscle, and iWAT of both *Sdhaf4* ^Alb^‐KO mice and ADF intervened mice (Figure 6m,n). Moreover, the cGMP level in target tissues changed coordinately with circulating NO level in *Sdhaf4* ^Alb^‐KO mice with or without L‐NAME intervention (Figure 6o–q). To further prove that enhanced tissue insulin sensitivity was a direct effect of NO, we employed a short‐term treatment of L‐NAME to inhibit production of NO, 24 h treatment of L‐NAME in *Sdhaf4* ^Alb^‐KO mice efficiently blocked the production of NO in liver and the circulating NO level in serum (Figure S9a,b, Supporting Information). More importantly, the improvement of insulin signaling in liver, adipose, and muscle was dramatically inhibited by addition of L‐NAME (Figure S9c–f, Supporting Information). Above all, we speculate that hepatic SDHAF4‐arginine axle drives the excessive production of NO which has autocrine action as well as targeting peripheral tissues for enhanced insulin sensitivity via cGMP signal.

### 2.6 Hepatic NOS3 Accounts for the Circulating NO in Metabolic Improved Mice

**Figure 7:**
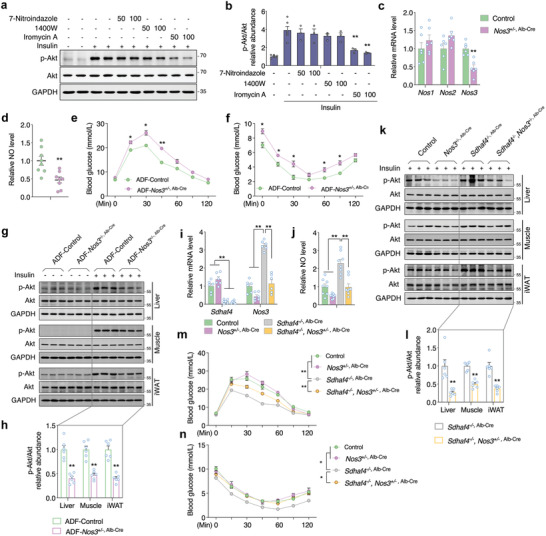
Hepatic NOS3 accounts for the circulating NO in metabolic improved mice. a,b) Immunoblots analysis of p‐Akt in C2C12 cells that co‐cultured with primary hepatocytes from *Sdhaf4* ^Alb^ KO mice followed with NOS inhibitors for 48 h and insulin challenge for 15 min, *n* = 6. c) mRNA levels of *Nos1*, *Nos2*, and *Nos3* in the liver of control and *Nos3* hepatic heterozygous knockout mice (*Nos3* ^+/−, Alb‐Cre^), *n* = 6. d) NO level in the serum of control and *Nos3* ^+/−, Alb‐Cre^ mice, *n* = 8. e) Glucose tolerance test and f) insulin tolerance test in the control and *Nos3* ^+/−, Alb‐Cre^ mice under regular feeding or ADF intervention for 4 weeks, *n* = 6. g) Immunoblots analysis of p‐Akt level in liver, muscle, and iWAT of control and *Nos3* ^+/−, Alb‐Cre^ mice under ADF intervention for 4 weeks with or without insulin challenge for 15 min, representative blotting images, h) summary analysis of arbitrary unit, *n* = 6. i) mRNA level of *Sdhaf4* and *Nos3* and j) serum NO level in the mice under hepatic *Sdhaf4* homozygous knockout and/or Nos3 heterozygous knockout, *n* = 6. k) Immunoblots analysis of p‐Akt level in liver, muscle, and iWAT of mice under hepatic *Sdhaf4* homozygous knockout and/or Nos3 heterozygous knockout followed by insulin challenge for 15 min, representative blotting images, l) summary analysis of arbitrary unit, *n* = 6. m) Glucose tolerance test and n) insulin tolerance test in mice under hepatic *Sdhaf4* homozygous knockout and/or Nos3 heterozygous knockout, *n* = 6. Values are mean ± SEM, *p < 0.05, **p < 0.01.

Since both NOS1 and NOS3 were found significantly increased in the liver of both mice model, we intended to determine the exact source of NO accounting for the metabolic benefits in mice. First, administration of specific NOS inhibitors (7‐Ni for NOS1, 1400W for NOS2, and Iromycin A for NOS3) to co‐cultured C2C12 myotubes with primary hepatocytes from *Sdhaf4* ^Alb^‐KO mice showed that inhibition of NOS3 effectively prohibited the Akt phosphorylation upon insulin challenge (**Figure** **7**a,b), suggesting that NOS3‐mediated NO production may account for the systemic metabolic improvement in *Sdhaf4* ^Alb^‐KO or ADF intervened mice. We then generated a hepatic heterozygous knockout of *Nos3* in mice (*Nos3* ^+/−, Alb‐Cre^), which presented 50% of *Nos3* expression in liver (Figure 7c) and circulating NO in serum (Figure 7d). Further ADF intervention on both *Nos3* ^+/−, Alb‐Cre^ and control mice revealed suppressed glucose and insulin tolerance in *Nos3* ^+/−, Alb‐Cre^ mice (Figure 7e,f). In addition, decreased Akt phosphorylation in response to insulin challenge was also observed in *Nos3* ^+/−, Alb‐Cre^ mice comparing control mice under ADF (Figure 7g,h). We further cross *Sdhaf4* ^Alb^‐KO (*Sdhaf4* ^–/−, Alb‐Cre^) mice with *Nos3* ^+/−, Alb‐Cre^ mice for *Sdhaf4* ^–/–^, *Nos3* ^+/−, Alb‐Cre^ mice which presented total loss of *Sdhaf4* and 50% expression of *Nos3* in liver (Figure 7i). As expected, *Sdhaf4* ^–/–^, *Nos3* ^+/−, Alb‐Cre^ mice had significant decreased circulating NO level comparing to *Sdhaf4* ^Alb^‐KO mice (Figure 7j). Meanwhile, knockdown of *Nos3* also dramatically decreased p‐Akt level in *Sdhaf4* ^Alb^‐KO mice in response to insulin challenge (Figure 7k,l). Oral glucose tolerance test (OGTT) and insulin tolerance test (ITT) analysis consistently revealed that glucose and insulin tolerance capacity in *Sdhaf4* ^Alb^‐KO mice were significantly suppressed by further knockdown of *Nos3* (Figure 7m,n). Taken together, our data indicate that hepatic NOS3 directly contributes the circulating NO in *Sdhaf4* ^Alb^‐KO and ADF intervened mice, and the SDHAF4‐arginine‐NO axle is one of the underlying mechanisms that regulating the metabolic benefits of ADF intervention.

### 2.7 Hepatic Overexpression of *Sdhaf4* Attenuates Benefits of ADF Mice on Insulin Sensitivity

**Figure 8:**
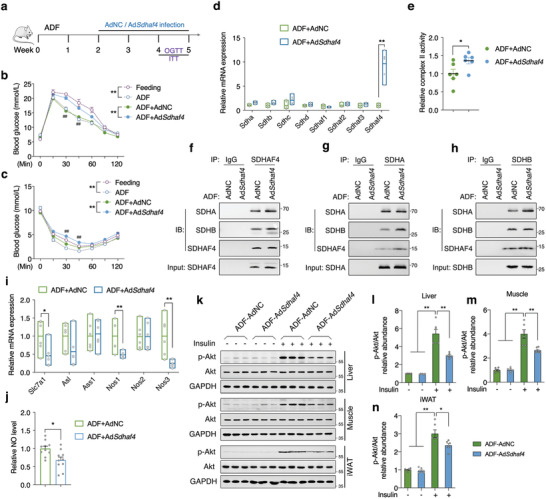
Hepatic overexpression of *Sdhaf4* attenuates benefits of ADF mice on insulin sensitivity. a) Representative scheme of ADF and adenovirus intervention study. b) Glucose tolerance test and c) insulin tolerance test in mice under regular feeding, or ADF, or ADF with adenovirus intervention, *n* = 6 for feeding and ADF groups, *n* = 12 for ADF+AdNC and ADF+Ad*Sdhaf4* groups. d) mRNA levels of *Sdha*, *Sdhb*, *Shdc*, *Sdhd*, *Sdhaf1*, *Sdhaf2*, *Sdhaf3*, and *Sdhaf4* in livers of ADF mice with AdNC and Ad*Sdhaf4* intervention, *n* = 6. e) Relative mitochondrial complex II activity in livers of ADF mice with AdNC and Ad*Sdhaf4* intervention, *n* = 6. f) Coimmunoprecipitation of SDHAF4 with SDHA/SDHB, g) SDHA with SDHB/SDHAF4, h) SDHB with SDHA/SDHAF4 from the lysates of ADF mice liver after AdNC and Ad*Sdhaf4* intervention, *n* = 3. i) mRNA levels of *Slc7a1*, *Asl*, *Ass1*, *Nos1*, *Nos2*, and *Nos3* in livers of ADF mice with AdNC and Ad*Sdhaf4* intervention, *n* = 6. j) Relative serum NO level of ADF mice with AdNC and Ad*Sdhaf4* intervention, *n* = 9. k–n) Immunoblots analysis of p‐Akt level in liver, muscle, and iWAT of ADF mice after AdNC or Ad*Sdhaf4* intervention with or without insulin challenge, *n* = 6. Values are mean ± SEM, *p < 0.05, **p < 0.01.

Above observations indicated that improved insulin sensitivity in mice under ADF intervention was primarily attributed to hepatic suppression of complex II assembly via SDHAF4 deficiency. To further verify such assumption, adenovirus‐based infection was employed for the transient expression *Sdhaf4* in liver during ADF process (**Figure** **8**a). Consistently, ADF intervention showed improved glucose tolerance and insulin sensitivity compared to regular feeding (Figure 8b,c), and mice infected with control vector (AdNC) during ADF showed comparable changes with none infection ADF mice, while mice infected with expression vector (Ad*Sdhaf4*) showed dramatically decreased glucose tolerance and insulin sensitivity compared to AdNC group during ADF (Figure 8b,c). mRNA expression further confirmed highly expressed *Sdhaf4* without affecting expression of other assembly factors and SDH subunits in ADF mice (Figure 8d). Moreover, overexpression of *Sdhaf4* dramatically improved complex II activity and SDH subunits assembly during ADF (Figure 8e–h). As expected, the induction of hepatic arginine‐NO cycle was suppressed as evidenced by decreased *Nos1*, *Nos3*, *Slc7a1* expression and serum NO level (Figure 8i,j), which were supposed to increase during ADF compared to regular feeding (Figure 6). Consistently, in vivo insulin challenge also confirmed that hepatic *Sdhaf4* overexpression sufficiently attenuated the improvement of systemic insulin sensitivity in ADF mice evidenced by decreased phosphorylation of Akt in liver, muscle, and iWAT tissues (Figure 8k–n). Taken together, these data reveal a highly dynamic and interactive mitochondria associated metabolism network in liver, which suggests that suppression of hepatic complex II assembly could be an intriguing avenue for improving metabolic capacity.

## 3 Discussion

CR has gained increasing attention over the past few decades as potential effective intervention for improvement of metabolic condition especially on weight management^[^ ^31^ ^]^ and many molecular mechanisms have been proposed. Recent studies indicate that daily fasting improves health and survival independent of diet composition and calories,^[^ ^4^ ^]^ suggesting a systemic metabolic reprogramming may account for the major effects of CR. As another popular form of dietary restriction, ADF is raising increasing intention as CR but with limited understanding. In the present study, we demonstrate that liver metabolic reprogramming plays crucial role in ADF‐mediated metabolic benefits. More intriguingly, instead of improving mitochondrial function, partial mitochondrial suppression due to lowered complex II assembly could trigger liver metabolic remodeling and systemic metabolic benefits under ADF, highlighting an intricate involvement of dynamic mitochondria in modulating metabolic condition.

Among many forms of dietary restriction, ADF is the most popular one involving 24 h feasting period with 24 h fasting cycle. Even though ADF and daily CR showed similar effect on body weight, ADF produces greater benefit of insulin sensitivity in insulin‐resistant participants than daily CR.^[^ ^32^ ^]^ Moreover, a randomized controlled trial study in healthy middle‐aged humans also revealed the physiological impact by modified ADF and supports its safety.^[^ ^13^ ^]^ Consistently, we found significant improved glucose and insulin tolerance in healthy adult male mice after 4 weeks ADF intervention (Figure 1). Given the vital role of liver in mediating glucose and amino acid metabolisms,^[^ ^33^, ^34^ ^]^ liver was thereby further investigated for the molecular changes.

Mitochondria are closely involved in liver metabolism. Though several studies have revealed multiple mitochondrial adaptions during different forms of CR,^[^ ^35^, ^36^, ^37^ ^]^ how liver mitochondria responding to specific ADF intervention has not been investigated. The metabolomics analysis of mouse liver after ADF led us to propose certain metabolic remodeling may occur in mitochondria, since most altered metabolites were primarily associated with amino acid metabolism (Figure 1). It was indeed unexpected to observe specifically decreased complex II activity in liver, given the general beneficial observations in animals and human studies with ADF intervention.^[^ ^38^ ^]^ However, in addition to electron transport chain, complex II is the only enzyme complex that involves in both TCA cycle and oxidative phosphorylation. We thereby propose that suppressed complex II may have more significant impact on TCA cycle in order to trigger compensatory network in liver, which we identified as SDHAF4‐arginine‐NO axle. Despite common acceptance of mitochondrial dysfunction in promoting diseases progression,^[^ ^39^, ^40^ ^]^ here we demonstrated that specific mitochondrial abnormality in liver could, however, activate compensatory pathway to improve systemic health. Whether this dynamic network occurs in other tissues warrants further investigation.

Among the five electron transport chain complexes, complex II is the simplest one with only four subunits (SDHA/B/C/D). Yet, functional complex II still requires sequential assembly with help of assembly factors.^[^ ^41^ ^]^ SDHAF2 was suggested to mediate the maturation of SDHA,^[^ ^25^ ^]^ which was further promoted to assemble with SDHB by SDHAF4 to initiate the assembly of complex II.^[^ ^26^, ^42^ ^]^ Thereby SDHAF2 and SDHAF4‐mediated activities are the key steps for functional complex II assembly. It is unexpected to observe decreased SDHAF4 and disrupted complex II assembly in the liver of ADF intervened mice, which we proposed as the key factor contributing to specific decrease of complex II activity (Figure 1). While, the detail mechanisms of ADF regulating hepatic SDHAF4 expression remain further investigation. Further hepatic knockout of *Sdhaf4* in mice demonstrated consistent metabolic benefits as ADF (Figures 2 and 3), indicating that SDHAF4‐mediated complex II suppression may be the leading factor that drives ADF benefits on glucose and insulin tolerance. Though SDH subunits and assembly factors are essential to functional complex II, their clinical mutations were associated with diverse phenotypes.^[^ ^43^, ^44^, ^45^ ^]^ As a newly identified assembly factor, the present study is the first to report the physiological involvement of SDHAF4 in regulating hepatic metabolic remodeling under ADF intervention.

CR is accepted as a decrease in calorie intake without malnutrition, while ADF is another dietary regimen without restriction of total calorie intake.^[^ ^32^ ^]^ It is well acknowledged that mTOR and AMPK signaling play vital role in regulating metabolic health under CR.^[^ ^46^ ^]^ Here, we reported that either mTOR or AMPK signaling was not altered in the liver by ADF or *Sdhaf4* KO, though that ATP levels were moderately decreased in both models (Figure S3, Supporting Information). Given the observations that mitochondrial number, mitophagy activity, as well as NAD^+^/NADH level remained unaffected, we thereby assumed that neither ADF nor *Sdhaf4* ^Alb^‐KO could provoke dramatic mitochondrial stress. Instead, liver in ADF or *Sdhaf4* ^Alb^‐KO mice presented primarily healthy metabolic reprogramming due to deficient SDH activity. Further bile acid profile, feces composition, and calorie assay supported a normal energy absorption/utilization in *Sdhaf4* ^Alb^‐KO mice (Figure S3, Supporting Information). Though the primary bile acid CDCA was found moderately decreased in the *Sdhaf4* ^Alb^‐KO mice, previous reports indicate that the addition of CDCA could have benefits on obesity.^[^ ^47^, ^48^ ^]^ Thus, we speculate that such level of CDCA decrease has neither positive nor negative impact on mice metabolism, and the improved metabolism benefits in *Sdhaf4* ^Alb^‐KO mice were primarily attributed to hepatic‐derived arginine‐NO axle.

Arginine is known to be involved in a number of biological processes including urea cycle and nitric oxide production.^[^ ^49^ ^]^ Metabolomics assay of both serum and liver tissues in *Sdhaf4* ^Alb^‐KO mice consistently identified arginine biosynthesis being the top affected pathway (Figures 5 and 6). As fumarate being byproduct of arginine biosynthesis cycle,^[^ ^28^, ^29^ ^]^ it is rational to conclude that arginine pathway was activated to mitigate the breakdown of TCA metabolism cycle, since disrupted complex II assembly could result in SDH subunits degradation and directly decrease fumarate level to suppress TCA metabolic capability (Figure 5). Either ADF or *Sdhaf4* ^Alb^‐KO mice exhibited obvious improvement on systemic insulin response assuming that liver may release certain circulating mediators, the assumption was consolidated by co‐culture of primary hepatocytes with myotubes and pre‐adipose cells (Figure 6). Citrulline and nitric oxide were thus instantly considered as candidates since they were increased in the serum of both ADF or *Sdhaf4* ^Alb^‐KO mice and have been reported to modulate insulin sensitivity.^[^ ^50^, ^51^ ^]^ While, addition of NOS inhibitors in vitro and in vivo further narrowed down to NOS3 overproduced nitric oxide being the key effector (Figure 6). This result is supported by direct knockdown of *Nos3* in WT or *Sdhaf4* ^Alb^‐KO mice, whose glucose and insulin tolerance were dramatically suppressed by the hepatic knockdown of *Nos3* (Figure 7). However, how NOS3 was induced in hepatocytes of *Sdhaf4* ^Alb^‐KO mice remains elusive, therefore, the molecular network responding to hepatic SDH deficiency should be further clarified.

As the most popular form of life intervention regimens, ADF has been suggested as a potential strategy for clinical intervention on obesity and diabetic populations. Yet, such form of intervention is quite challenging and difficult to follow for long‐term purpose. Hence, the exploration of molecular networks underpinning ADF benefits is greatly needed for precise and more acceptable strategy. Our collected data reveal a novel and convincing link between complex II assembly with arginine metabolism, and for the first time showing that suppressed hepatic complex II activity presents long‐term systemic metabolic benefits via activation of arginine biosynthesis. At the meantime, our study demonstrates that hepatic SDHAF4‐arginine‐NO may be the proximal axle that drives metabolic benefits of ADF intervention, providing further understanding of dietary restriction‐associated metabolic remodeling. Overall, the study indicates that the benefits of ADF may be from the suppression on mitochondrial complex II activity by decrease of SDHAF4 and that modulation of hepatic SDHAF4 level could be a possible approach for managing insulin sensitivity and related metabolic disorders.

## 4 Experimental Section

### Antibodies and Reagents

Cell culture medium was purchased from Life Technologies (San Diego, CA). Collagenase VI (DY40128) was obtained from DiYi Biochemical Inc., Ltd. (Shanghai, China). The Insulin enzyme‐linked immunosorbent assay was obtained from Life Technologies (San Diego, CA, No. EMINS). Nitric oxide assay kits were purchased from BioVision Inc. (San Francisco, CA). Antibodies against phospho‐AKT (4060), AKT (4691), GAPDH (5174), SDHAF2 (45849), SDHA (11998), LC3B (3868), Phospho‐FoxO1 (Ser256) (84192), FoxO1 (2880), PGC‐1*α* (2178), Phospho‐AMPK*α* (Thr172) (50081), AMPK*α* (5831), Phospho‐mTOR (Ser2448) (5536), and mTOR (2983) were obtained from Cell Signaling Technology (Danvers, MA). Antibodies against SDHB (178423), SDHAF4 (122196), SDHC (155999), and SDHD (189945) were purchased from Abcam (Cambridge, UK). Antibodies against ubiquitin‐HRP (8017), SQSTM1 (28359), Parkin (32282), PINK1 (528052), NOS1 (5302), NOS2 (7271), and NOS3 (376751) were purchased from Santa Cruz Biotechnology (Santa Cruz, CA). Other reagents used in this study were purchased from Sigma (St. Louis, MO).

### Animals

All animals were maintained and used in accordance with the guidelines of the Institutional Animal Care and Use Committee of Xi'an Jiaotong University. The protocol was approved by the Animal Care and Use Committee of the School of Life Science and Technology, Xi'an Jiaotong University (no. 2017‐0016).

For the ADF schedule, male C57BL/6J mice at 8 weeks of age were randomly divided into ad libitum fed group, or a 24 h fasting/24 h feeding group. The fasted mice were placed in new cages without food after 24 h feeding period to prevent eating remnants of chow diet on the cage floor. All mice were individually housed 1 week prior to harvesting tissue.

*Generation of hepatic knockout mice*: The *Sdhaf4* ^flox^ mice were generated by Beijing Biocytogen Co., Ltd. (Beijing, China). A loxP strategy was used to target the *Sdhaf4* locus in order to generate *Sdhaf4* deficient mice. The exon2 was flanked by a pair of loxP sites and deleted upon Cre‐loxP‐mediated recombination. The *Sdhaf4* ^flox^ mice were detected by PCR amplification and direct sequencing, and further confirmed by southern blotting. The *Nos3* ^flox^ mice were generated by Cyagen Biosciences Inc. (Jiangsu, China). The exon2‐4 was flanked by a pair of loxP sites and deleted upon Cre‐loxP‐mediated recombination. Alb‐Cre mice were obtained from the Jackson Laboratory (Bar Harbor, ME, no. 003574). Hepatic homozygous knockout mice of *Sdhaf4* (*Sdhaf4* ^Alb^‐KO) were generated by cross Alb‐Cre mice with *Sdhaf4* ^flox/flox^ mice. *Sdhaf4* ^Alb^‐KO mice were born at the expected Mendelian ratios and showed normal fertility.

For the adenovirus‐mediated rescue expression of SDHAF4 in mouse liver, 8 weeks old liver specific knockout male mice were used. The mouse *Sdhaf4* sequence was inserted into a pENTR entry vector followed by recombination with pAd/CMV/V5‐DEST vector (the Gateway adenoviral expression system was utilized in these experiments). Adenoviruses and control adenovirus were packaged in 293A cells and purified with ultracentrifugation. The viruses were tittered and administrated via caudal vein injection (5 × 10^8^ plaque‐forming units of virus per mouse) to *Sdhaf4* ^Alb^‐KO mice at the age of 8 weeks. 2 weeks after injection, mice were sacrificed to collect the tissues and plasma for analyses.

### Glucose and Insulin Tolerance Tests

GTT and ITT were performed following previously published study.^[^ ^52^ ^]^ For GTT, mice were fasted overnight and administered glucose (2 g kg^−1^), blood glucose levels were measured at 0, 15, 30, 45, 60, 90, and 120 min. For ITT, mice were fasted for 6 h and administered insulin intraperitoneally (0.7 U kg^−1^ for chow diet and 0.75 U kg^−1^ for HFD), blood glucose was measured at 0, 15, 30, 45, 60, 90, and 120 min.

### ATP Assay

The level of total liver ATP was measured by an ATP assay kit (Beyotime, Jiangsu, China) following manufacturer's instructions. Briefly, fresh liver tissue was lysed with lysate in the kit, collecting the supernatant by centrifugation at 10 000*g* for 10 min at 4 ℃. The supernatant was mixed with luciferase reagent and the emitted light was measured with a microplate photometer. Protein concentration was quantified by BCA kit and total ATP levels were normalized by protein concentration.

### cGMP Assay

The level of cGMP was measured by cGMP assay kit (Jiancheng Bio, Nanjing, China) following manufacturer's instructions. The kit applied competition method to detect the content of cGMP. Briefly, the fresh tissue was placed into buffer at 1:9 (iWAT at 1:4) by weight/volume, fully homogenized at 4 ℃, centrifuged at 3000 rpm for 20 min at 4 ℃, and the supernatant was collected for cGMP detection. Protein concentration was quantified by BCA kit and cGMP levels were normalized by protein concentration.

*NADH assay*: The detection of NADH in tissues was based on the chromogenic reaction of WST‐8 using assay kit (Beyotime, Jiangsu, China). 400 µL NADH extraction buffer was added to 30 mg fresh liver sample to be homogenized on ice. Centrifugation was performed at 12 000*g* at 4 ℃ for 10 min, the supernatant was collected for analysis following manufacturer's instructions.

### TC and TG Assay

Total triglycerides in liver were measured using a GPO‐PAP enzyme triglyceride detection kit (Jiancheng Bio, Nanjing, China), and total cholesterol were measured using a COD‐PAP enzyme cholesterol detection kit (Jiancheng Bio, Nanjing, China) following manufacturer's instructions. The protein concentration was detected by bicinchoninic acid (BCA) method, and the sample was modulated to a uniform concentration. The content of triglycerides and cholesterol was calculated according to the standard curve.

### Glycogen Assay

The liver glycogen detection was performed with commercial liver glycogen detection ELISA kit (Jiancheng Bio, Nanjing, China). Briefly, fresh liver tissues were homogenized in pre‐cooled phosphate‐buffered saline buffer solution and centrifuged at 1000*g *at 4 ℃ for supernatant collection. Protein concentration was detected by BCA method, and the protein concentration of sample was adjusted to the agreed concentration. Enzyme‐linked immunoassay (ELISA) was performed according to manufacturer's instructions in the kit.

### Analysis of Residue Energy in Feces

The residual energy in feces was detected with freshly collected mice feces stored in liquid nitrogen. Samples were sent to Qingdao Standard Testing Co., LTD. (Qingdao, China) for analysis. The tests were conducted according to GB‐2016 National Food safety standard. Carbohydrates content (per 100 g) = 100 – protein – fat – water – Ash. When the nutrient content of fat, protein, and carbohydrate ≤0.5 g/100 g, it was labeled as “0”.

### Bile Acids Profile

Bile acids (BAs) profile in liver was performed on Ultra performance liquid chromatography‐tandem mass spectrometer (UPLC‐MS/MS) platform (Metabo‐Profile, Shanghai, China). All BAs standards were synthesized by Metabo‐Profile lab or obtained from Steraloids Inc. (Newport, RI, USA) and TRC Chemicals (Toronto, ON, Canada). The raw data files generated by UPLC‐MS/MS were processed using the QuanMET software (v2.0, Metabo‐Profile, Shanghai, China) to perform peak integration, calibration, and quantitation for each metabolite. The powerful package R studio was used for statistical analyses.

### Grip Strength Test

The grip strength was tested by the apparatus of Grip‐strength meter (Ugo basile, Italy) according to the manufacturer's instructions. Briefly, each mouse was held by the tail and lowered toward the dynamometer's triangle and allowed to grasp it with its forepaws, then pulled the mouse steadily by the tail away from the rod until the mouse's grip was broken. The apparatus recorded the peak value of strength. The grip strength of each mouse was tested for three times, each time interval was 5 min, and the maximum value was used as the grip strength score for that mouse.

### Open Filed Test

The open filed test was employed by a 42 × 42 × 42 cm polyvinyl chloride box with a camera monitoring the movement into and around the central and peripheral areas of the box. Mice were acclimatized 7 days before the experiment. Each mouse was placed onto the same corner zone of the open field box and the mouse was allowed to explore the test area for 15 min. The route was recorded and the results were analyzed (Anymaze, Stoelting, IL).

### Cell Culture

For primary hepatocytes: mice at age of 6 weeks were anesthetized and perfused through the hepatic portal vein with Buffer I (Hanks’ buffer, 2 × 10^−3^ m ethylene glycol tetraacetic acid, 0.1% glucose, pH 7.4) followed by Buffer II (Hanks’ buffer, 5 × 10^−3^ m CaCl_2_, 0.1% glucose, 0.6 mg mL^−1^ collagenase IV, pH 7.4). Livers were gently excised and shaken to release hepatic cells in ice‐cold Hanks’ buffer. The cells were further dispersed through a 25 mL pipette. The cell suspension was filtered through a 70 µm nylon cell‐strainer and the cells were left to settle for 10 min. The settled cells were diluted in 20 mL of ice‐cold hepatocyte‐washing medium (HWM) (standard WE medium supplemented with fetal bovine serum (FBS, 7% v/v), antibiotic, antimycotic solution (10 mL L^−1^)) and centrifuged at 50 *g* for 2 min at 4 °C. The supernatant was decanted and the cells were resuspended very gently in 20 mL of ice‐cold HWM. The cells were resuspended after washing twice with HWM. Viable hepatocytes (1 mL of 5 × 106 cells mL^−1^) were dispensed into each well of a collagen‐coated 12‐well culture plate. The cells were incubated at 37 °C with 5% CO_2_ for 2–3 h. The medium and dead or unattached cells were removed. Fresh hepatocyte‐culture medium (HCM) (standard William's medium E supplemented with FBS (7% v/v), insulin (10 mg L^−1^), sodium selenite (6.7 µg L^−1^), transferrin (5.5 mg L^−1^), sodium pyruvate (110 mg L^−1^), antibiotic, antimycotic solution (10 mL L^−1^) was added and the final concentration of 30 × 10^−3^ m sodium pyruvate and 5 × 10^−9^ m dexamethasone) was used in further experimental work.

*C2C12 and 3T3‐L1*: All cells were grown in a monolayer at 37 °C with 5% CO_2_. C2C12 (ATCC CRL‐1772) and 3T3‐L1 (ATCC CL‐173) cells were cultured in DMEM supplemented with 10% FBS, 100 units mL^−1^ penicillin, and 100 µg mL^−1^ streptomycin sulfate. For adipocytes differentiation, 3T3‐L1 cells were seeded at 3 × 10^5^ cm^−2^ on transwell grown to confluency. 2 days after confluency, cells were changed to differentiation medium (DMEM containing 10% FBS, 1 × 10^−6^ m dexamethasone, 1 ug mL^−1^ insulin, and 0.5 × 10^−3^ m isobutylmethylxanthine) for 2 days. Cells were maintained in adipocyte maintenance medium (DMEM containing 10% FBS and 1 ug mL^−1^ insulin) for another 4 days. Induced adipocytes were washed twice with DMEM and cultured in DMEM for another 6 h before co‐culture with hepatocytes. For myotubes differentiation, C2C12 cells were seeded at 3 × 10^5^ cm^−2^ on transwell and grown to confluency. Cells were then changed to differentiation medium (DMEM contained 2% horse serum) for 5 days. Myotubes were washed twice with DMEM and cultured in DMEM for another 6 h before co‐culture with hepatocytes.

### Mitochondrial Isolation and Measurement of Complexes Activity

Mitochondria were extracted from the fresh liver tissue. Briefly, 100 mg tissue was cut with scissors in mitochondria extraction buffer (0.25 m sucrose, 10 × 10^−3^ m Tris‐base, 1 × 10^−3^ m EDTA·2Na, pH 7.5). The buffer which contained tissue pieces was transferred to Dounce Tissue Grinders and was grinded 40 times gently. Cell debris was removed by low centrifugation, the supernatant was collected and centrifuged at 12 000*g*, 15 min, at 4 °C. The precipitate was washed twice by mitochondria extraction buffer before resuspending. Mitochondrial protein concentration was determined by BCA Protein Assay Kit (Thermo Scientific, no. 23229). Assays for complex I, complex II, complex III, complex IV, and complex V activities were performed according to methods previously described.^[^ ^53^ ^]^

### Histology

Small pieces of liver and adipose tissues were fixed in 4% paraformaldehyde, minced into 3–5 µm of thickness and stained with H&E or PAS for glycogen. Histological images were observed using an Olympus BX71 microscope.

### Metabolic Cage Analysis

Indirect calorimetry was performed with negative‐flow system cages Oxymax/CLAMS (Columbus Instruments) according to the manufacturer's instructions. Feeding and lighting conditions in metabolic cages were maintained consistent with those in the normal cages. Mice were allowed to acclimate for 24 h to minimize stress.

### Metabolomics

Liver samples were collected following standard procedure. Fasting serum were collected on the same day as liver and were stored at −80 °C. The untargeted metabolomics profiling was performed on XploreMET platform (Metabo‐Profile, Shanghai, China), following previously published methods.^[^ ^54^ ^]^

### Protein Extraction and Western Blot

Whole cell extracts of cells and tissues were obtained as follows: frozen cell pellets or tissues were homogenized in modified lysis buffer (20 × 10^−3^ m Tris (pH 7.5), 150 × 10^−3^ m NaCl, 1% Triton X‐100, and 1 × 10^−3^ m PMSF) supplemented with protease inhibitor cocktail (Sigma, St. Louis, MO), 1 × 10^−3^ m DTT, 20 × 10^−3^ m NaF, 1 × 10^−3^ m sodium orthovanadate, 10 × 10^−3^ m nicotinamide, 330 × 10^−9^ m Trichostatin A (Sigma, St. Louis, MO, no. T8552). Samples were sonicated and centrifuged at 15 000*g* for 15 min at 4 ℃. Supernatants were collected as whole lysates. Protein concentration was determined using BCA protein assay kit (Thermo Scientific, Waltham, MA, no. 23229). 10–20 µg protein samples were separated by 10% sodium dodecyl sulfate–polyacrylamide gel electrophoresis, transferred to pure nitrocellulose membranes (PerkinElmer Life Science, Boston, MA), followed by standard immunoblotting procedures and analysis. The blots were developed with autoradiography films (Clinx Science Instruments, Shanghai, China). The bands densitometry was analyzed through Clinx chemi analysis software.

### Coimmunoprecipitation Assay

Tissue lysates were collected in lysis buffer (20 × 10^−3^ m Tris (pH 7.5), 150 × 10^−3^ m NaCl, 1% Triton X‐100, and 1 × 10^−3^ m PMSF). SDHA, SDHB, ubiquitin protein were immunoprecipitated using anti‐SDHA antibody (5839, CST), SDHB antibody (178423, Abcam), and Ubiquitin antibody (8017, Santa Cruz, City, CA), respectively. SDHA, SDHB, and ubiquitin were western blotted using anti‐SDHA antibody (390381, Santa Cruz), anti‐SDHB antibody (271548, Santa Cruz), and anti‐ubiquitin‐HRP (8017, Santa Cruz).

### Extraction of mRNA and qPCR

Total RNA from either cultured cells or tissues were prepared using TRIzol reagents (Invitrogen, Carlsbad, CA), and reverse‐transcribed into cDNA with iScript cDNA synthesis kit (Bio‐Rad Laboratories, Hercules, CA), followed by qPCR using target‐specific primers in BIO‐RAD CFX96 qPCR Systems according to the manufacturer's protocol. All reactions were performed in triplicate, and relative amounts of mRNA were calculated using the comparative CT method. GAPDH was used as the control. Values showed were the amount of mRNA relative to the control group, which was arbitrarily defined as 1. Primer sequences are listed in Table S1 in the Supporting Information.

### Blood Analysis

Blood glucose levels were determined by ACCU‐CHEK Sensor Comfort Test Strips. Serum NO levels were determined by nitrite/nitrate Assay Kit following manufacture's instruction. Serum insulin was determined by Mouse Insulin ELISA Kit (Invitrogen, City, State) following manufacturer's instruction. Serum total cholesterol, triglyceride, creatinine, HDL, LDL, urea, ALT, and AST levels were determined by HITACHI automatic analyzer (Chiyoda, Japan).

### Statistical Analysis

Values were presented as mean ± SEM. Data were analyzed with Prism (GraphPad). Pairwise comparisons were analyzed using two‐tailed Student's *t* test. Other data were analyzed using one‐way or two‐way analysis of variance with correction for multiple comparisons. In all cases, *p* < 0.05 was considered significant.

## Conflict of Interest

The authors declare no conflict of interest.

## Author Contributions

X.W., W.L., and J.X. contributed equally to this work. J.L., Z.F., and A.Z. conceived the study, Z.F., J.L., Y.S., and J.L. designed the experiments; X.W., M.Z., and J.X. performed the most experiments with the help of A.Z., W.L., K.C., Y.C., X.W., H.L., F.Z., and M.Y.; X.W. and Z.F. analyzed the data; X.W., Z.F., and J.L. wrote the manuscript, J.L. and Z.F. supervised the study.

## Supporting information

Supporting Information

## Data Availability

The data that support the findings of this study are available in the supplementary material of this article.
