# Supplementary material for: Hepatic Suppression of Mitochondrial Complex II Assembly Drives Systemic Metabolic Benefits
Source: Adv Sci (Weinh). 2022 Jan 17;9(9):2105587. doi: 10.1002/advs.202105587 (PMC8948583; doi:10.1002/advs.202105587)
Supplement: Supplementary file 1 — Supporting Information [file ADVS-9-2105587-s001.pdf]

ADVANCED SCIENCE
Open Access

Supporting Information

for *Adv. Sci.,* DOI: 10.1002/advs.202105587

Hepatic suppression of mitochondrial complex II assembly drives systemic metabolic benefits

Xueqiang Wang, Weiqiang Lv, Jie Xu, Adi Zheng, Mengqi Zeng, Ke Cao, Xun Wang, Yuting Cui, Hao Li, Meng Yang, Yongping Shao, Fang Zhang, Xuan Zou, Jiangang Long, Zhihui Feng, Jiankang Liu**

Supporting Information

Hepatic suppression of mitochondrial complex II assembly drives systemic metabolic benefits

Xueqiang Wang, Weiqiang Lv, Jie Xu, Adi Zheng, Mengqi Zeng, Ke Cao, Xun Wang, Yuting Cui, Hao Li, Meng Yang, Yongping Shao, Fang Zhang, Xuan Zou, Jiangang Long, Zhihui Feng, Jiankang Liu**

Supplemental Figure 1

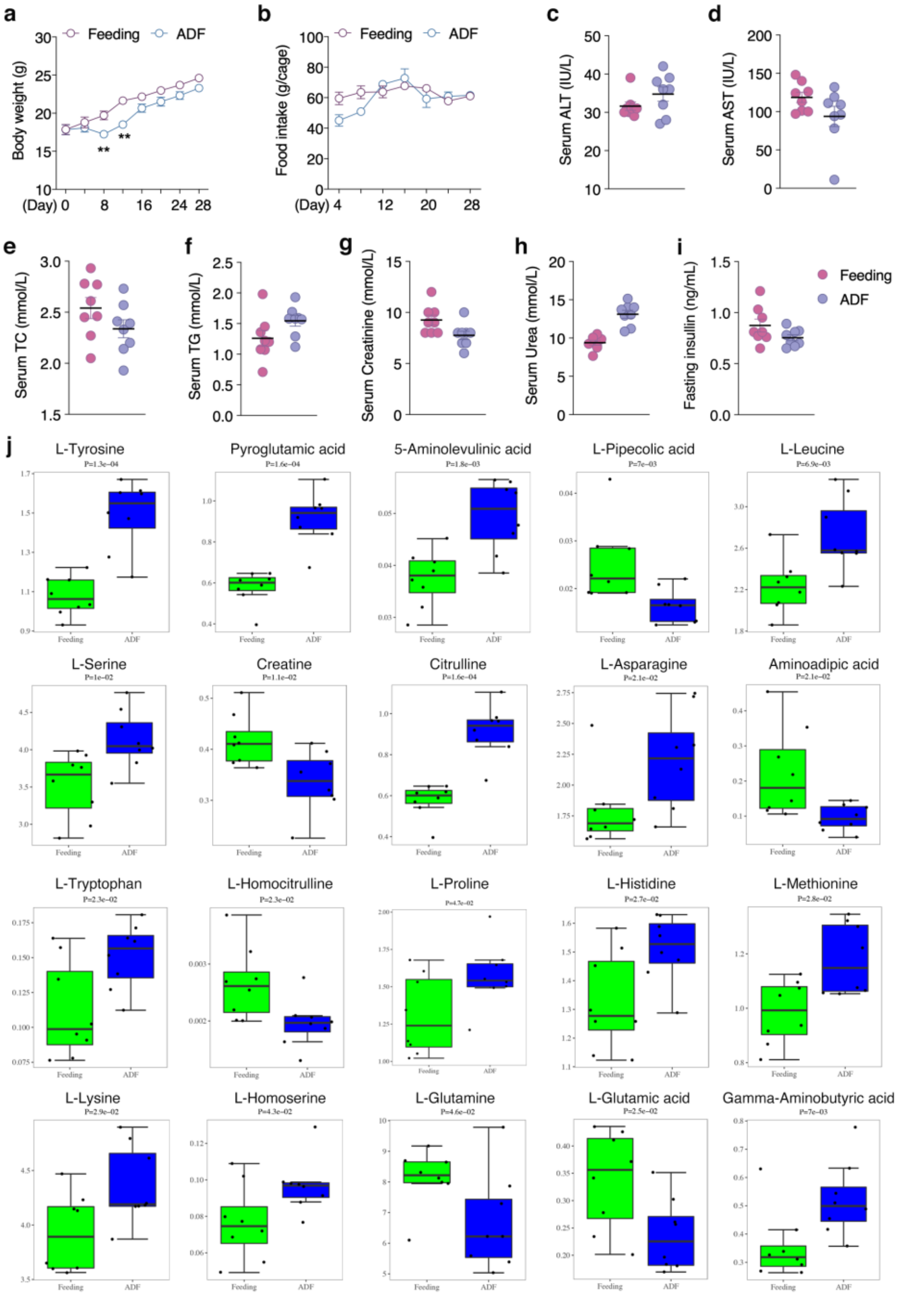

Figure S1 (Related to Figure 1). Blood analysis of mice under ADF intervention. a-b, Body weight curve (**a**) and food intake (**b**) during ADF intervention for 4 weeks, n=8. **c-i**, serum ALT, AST, total cholesterol, total glycerides, creatinine, urea and fasting insulin levels of mice after ADF intervention, n=8. **j**, Boxplots of significant amino acids metabolites differed after ADF intervention ($p < 0.05$). Values are mean ± SEM, *$p < 0.05$, **$p < 0.01$.

Supplemental Figure 2

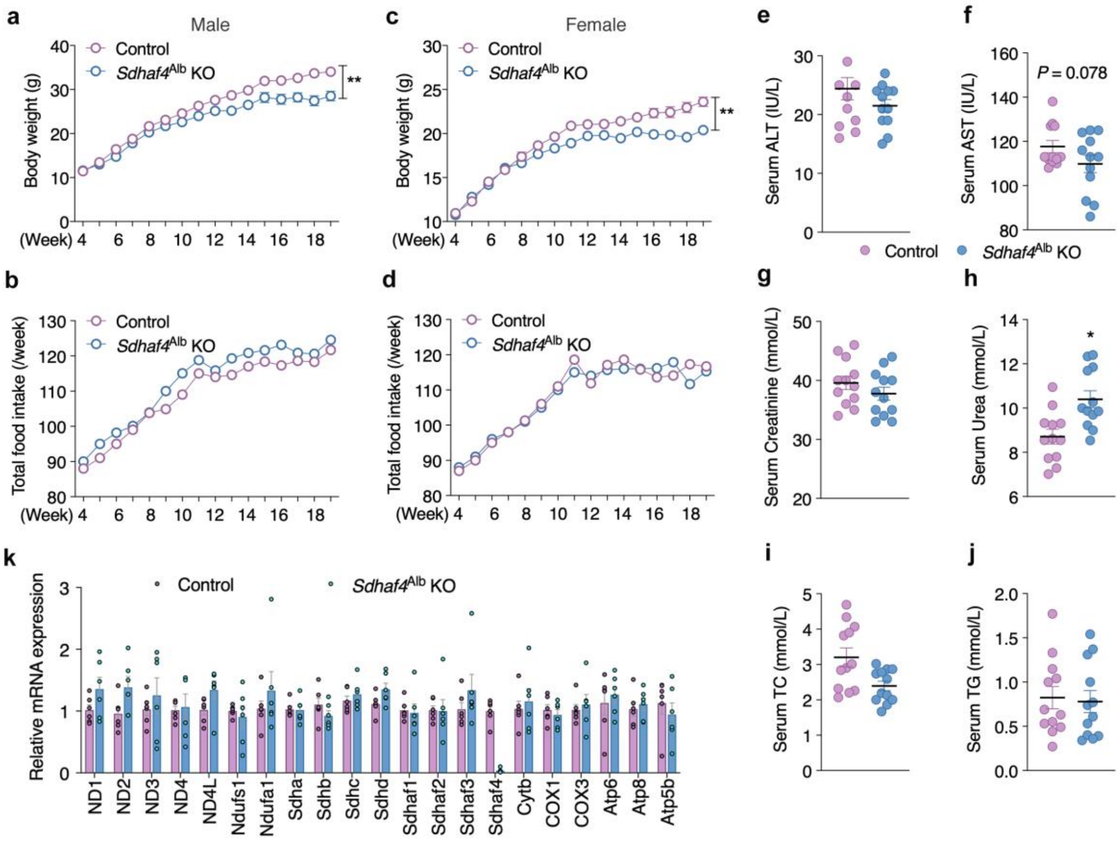

Figure S2 (Related to Figure 2). Characterization of hepatic *Sdhaf4* knockout mice. a-b, Body weight curve for both male (**a**) and female (**b**) control and *Sdhaf4*^{Alb} KO mice from age of 4 weeks to 19 weeks under normal feeding, n=9. **c-d**, Food intake curve for both male (**c**) and female (**d**) mice from age of 4 weeks to 19 weeks under normal feeding, n=9. **e-j**, Serum ALT (**e**), AST (**f**), creatinine (**g**), urea (**h**), total glycerides (**i**) and cholesterol (**j**) levels of control and *Sdhaf4*^{Alb} KO mice at the age of 8 weeks, n=9. **k**, mRNA levels of representive mitochondrial complex subunits in liver of control and *Sdhaf4*^{Alb} KO mice, n=9. Values are mean ± SEM, *p< 0.05, **p< 0.01.

Supplemental Figure 3

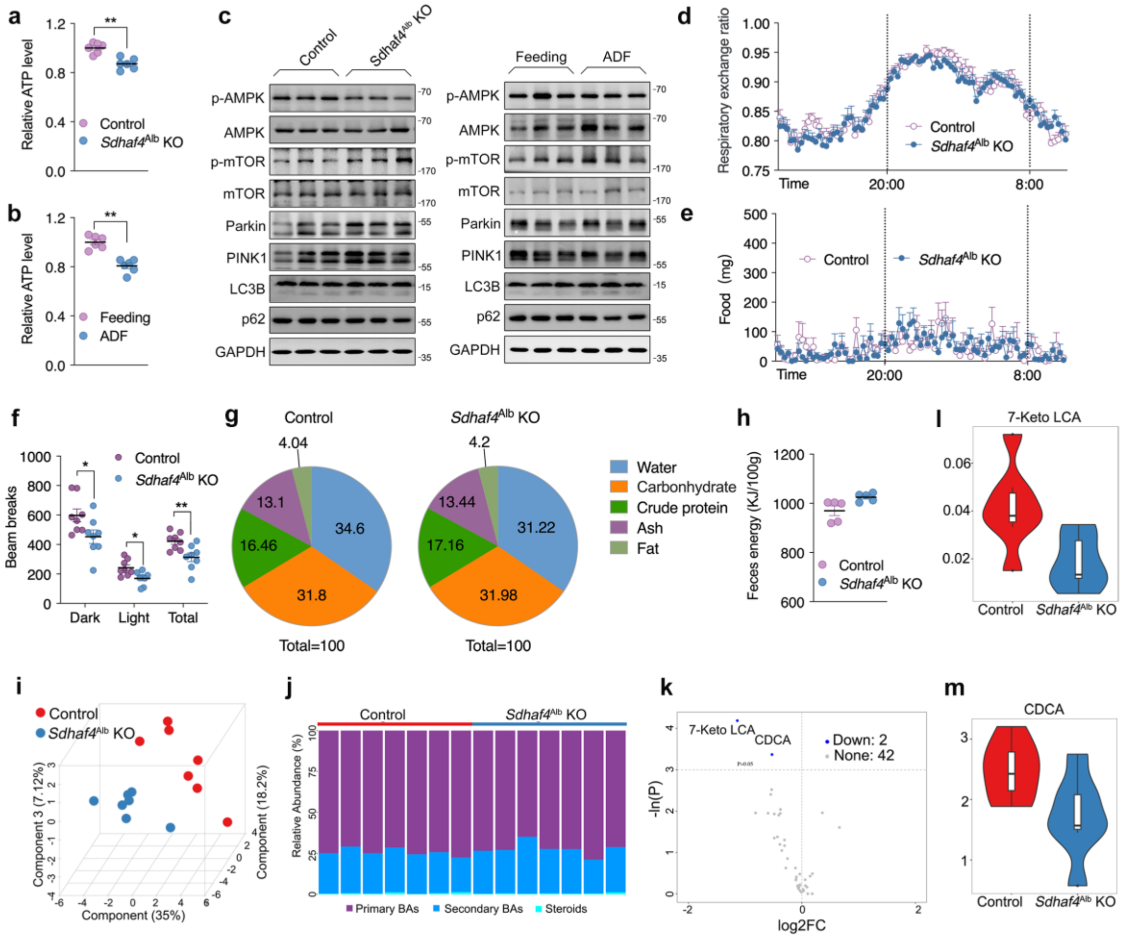

Figure S3 (Related to Figure 2). Hepatic *Sdhaf4* knockout mice present comparable changes in hepatic mitophagy, energy consumption, and bile acid metabolism. a-b, ATP level in the liver of control and *Sdhaf4*^{Alb} KO mice (**a**), regular feeding and ADF intervened mice for 4 weeks (**b**), n=6. **c**, Immunoblots analysis of mitophagy associated genes in control and *Sdhaf4*^{Alb} KO mice, regular feeding and ADF intervened mice for 4 weeks, n=3. **d-f**, Metabolic cage analysis of control and *Sdhaf4*^{Alb} KO mice at the age of 8 weeks, respiratory exchange ratio (**l**), food intake (**m**), and ambulatory activity (**n**) for 24 hours, n=8. **g-h**, Feces composition (**g**) and residue engery (**g**) in control and *Sdhaf4*^{Alb} KO mice, n=5. **i-k**, Profile analysis of bile acids in control and *Sdhaf4*^{Alb} KO mice, 3D PLS-DA scores plot (i), stacked chart figure for relative abundance of each metabolite classes (j), and the volcano plot showing the differential metabolites (k), n=7. **l-m**, Violin plot of differential metabolites 7-Keto LCA (**l**) and CDCA (**m**), n=7. Values are mean ± SEM, *p< 0.05, **p< 0.01. 7-Keto LCA, 7-keto Lithocholic Acid; CDCA, Chenodeoxycholic acid.

Supplemental Figure 4

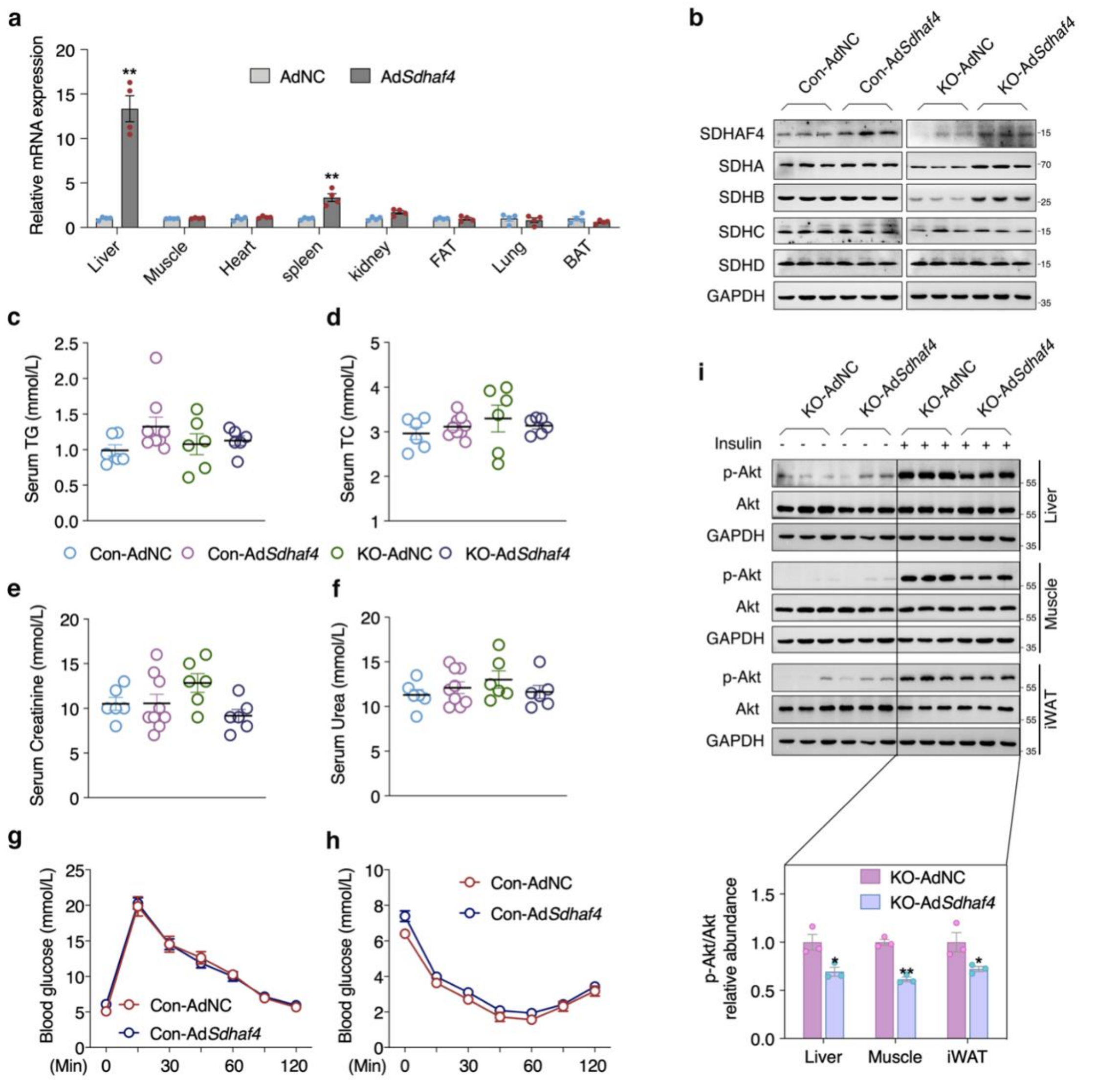

Figure S4 (Related to Figure 3). Hepatic rescue of *Sdhaf4* expression in knockout mice suppresses metabolic sensitivity. a, mRNA levels of *Sdhaf4* in tissues of control mice after adenovirus infection for 3 weeks, n=4. **b,** Immunoblots analysis of SDHAF4 and complex II subunits in control and *Sdhaf4*^{Alb} KO mice after adenovirus mediated hepatic *Sdhaf4* overexpression for 3 weeks, n=3. **c-f**, serum total glycerides (**c**), total cholesterol (**d**), creatinine (**e**), and urea (**f**) levels of control and *Sdhaf4*^{Alb} KO mice after hepatic *Sdhaf4* overexpression for 3 weeks, n=6 or 8. **g-h**, Glucose tolerance test (**g**) and insulin tolerance test (**h**) in control mice after adenovirus mediated hepatic *Sdhaf4* overexpression for 2 weeks, n=6, or 8. **i**, Immunoblots analysis of p-Akt level in iWAT, muscle, and liver tissues of control and *Sdhaf4*^{Alb} KO mice under adenovirus infection with or without insulin challenge, n=3. Values are mean ± SEM, *p< 0.05, **p< 0.01.

Supplemental Figure 5

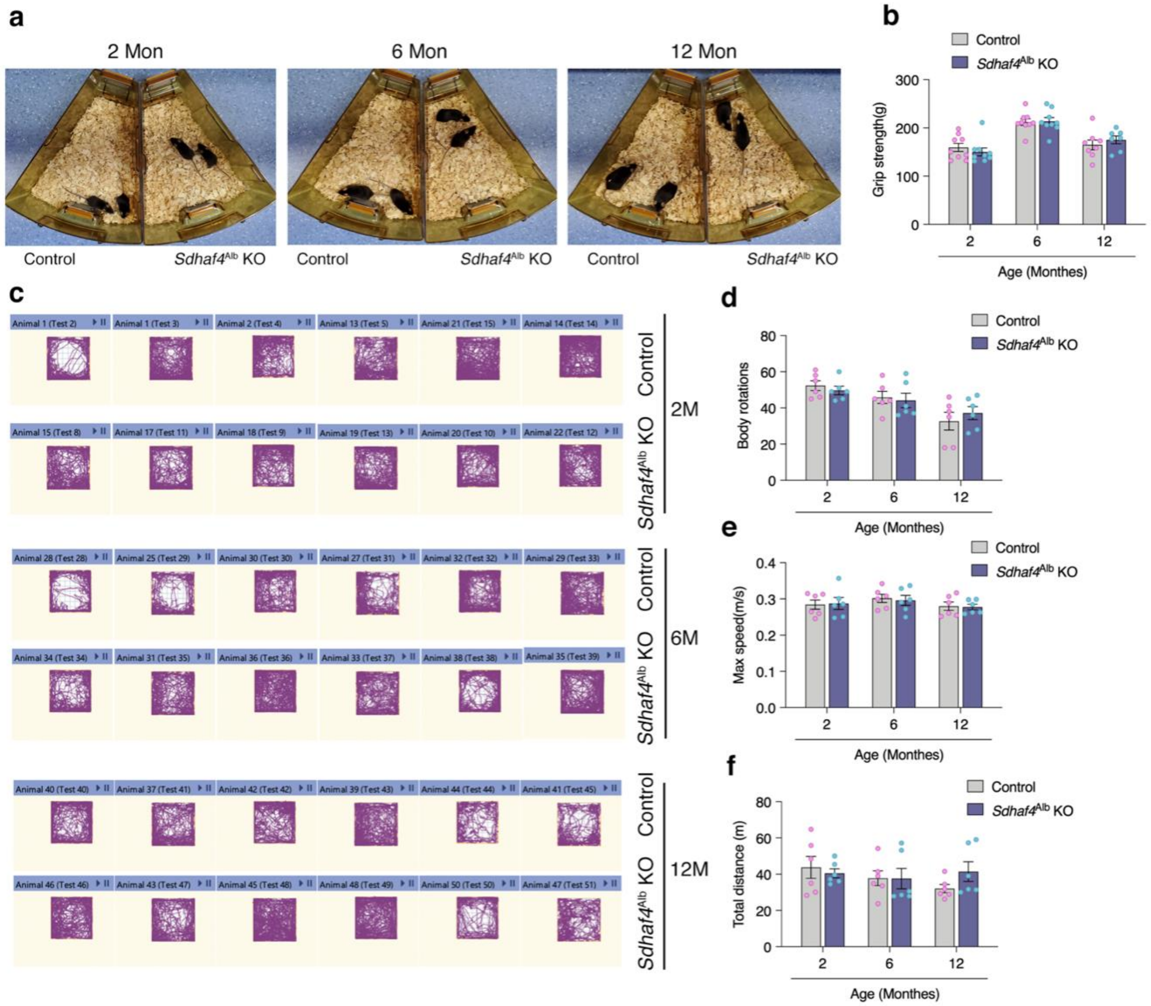

Figure S5 (Related to Figure 3). Aging performance of hepatic *Sdhaf4* knockout mice. a, Representative images of control and *Sdhaf4*^{Alb} KO mice at the age of different ages. **b**, Grip strength analysis of control and *Sdhaf4*^{Alb} KO mice at the age of different ages, n=9. **c-f**, Open filed test of control and *Sdhaf4*^{Alb} KO mice at the age of different ages, trace image of mice activity (**c**), body rotation records (**d**), max speed travelled (**e**), and total distance travelled (**f**), n=6. Values are mean ± SEM.

Supplemental Figure 6

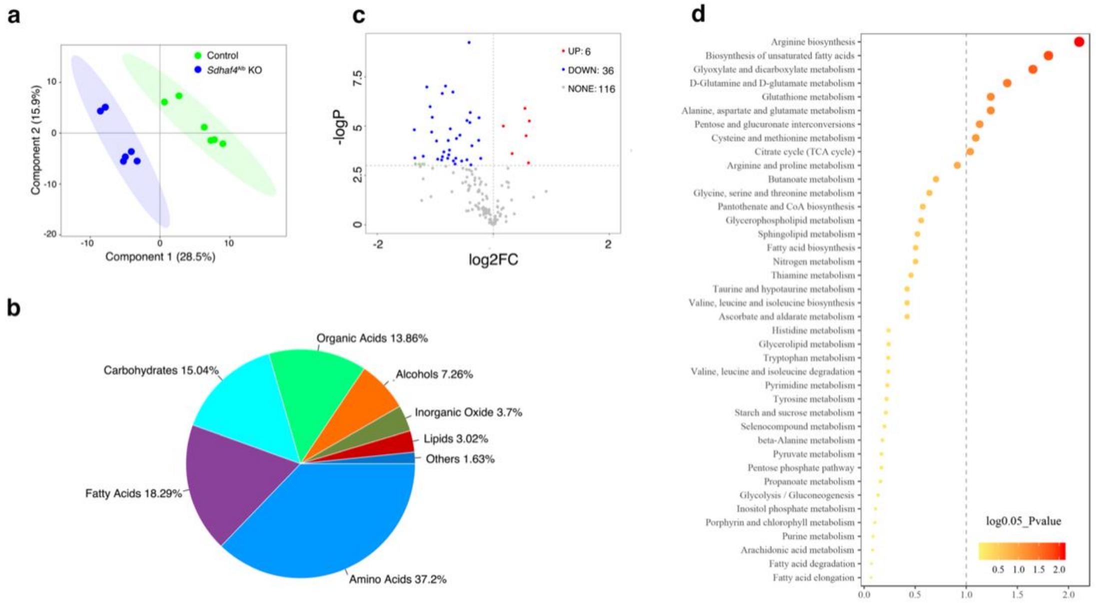

Figure S6 (Related to Figure 6). Metabolomics analysis for serums of control and *Sdhaf4*^{Alb} KO mice. a, 2D PLS-DA scores plot revealing classifications of the samples. **b**, Metabolite classes and compositions. **c**, Enhanced volcano plot showing the differential metabolites. **d**, Metabolic pathway enrichment analysis, n=6.

Supplemental Figure 7

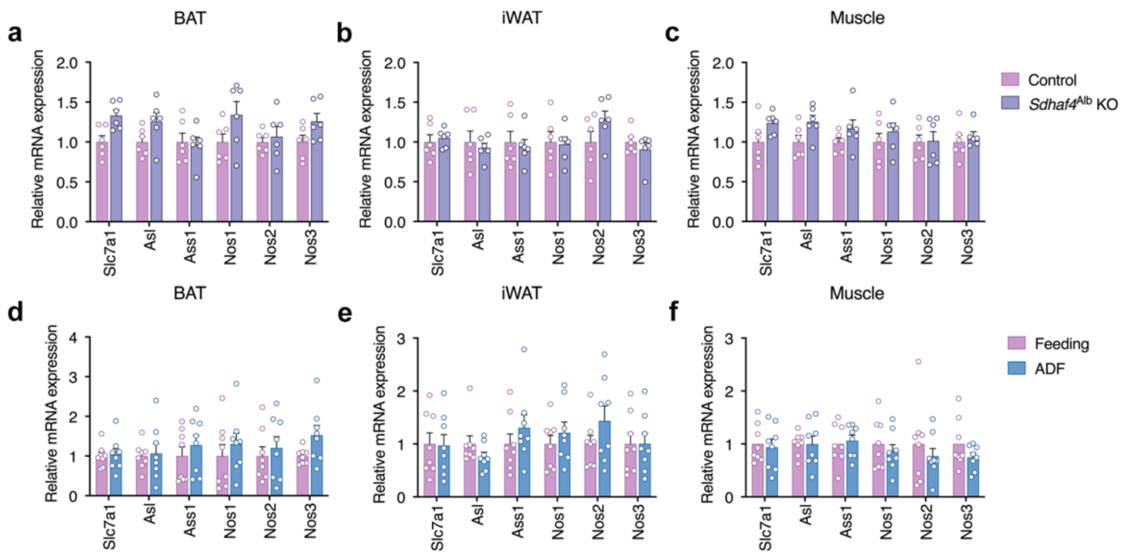

Figure S7(Related to Figure 6). NO synthesis in peripheral tissues of *Sdhaf4*^{Alb} KO mice and ADF intervened mice. a-c, mRNA levels of NO synthesis related genes in the BAT (**a**), iWAT (**b**), and muscle (**c**) tissues of control and *Sdhaf4*^{Alb} KO mice, n=6. **e-f**, mRNA levels of NO synthesis related genes in the BAT (**d**), iWAT(**e**), and muscle (**f**) tissues of mice under regular feeding or ADF intervention for 8 weeks, n=8. Values are mean ± SEM, *p< 0.05, **p< 0.01.

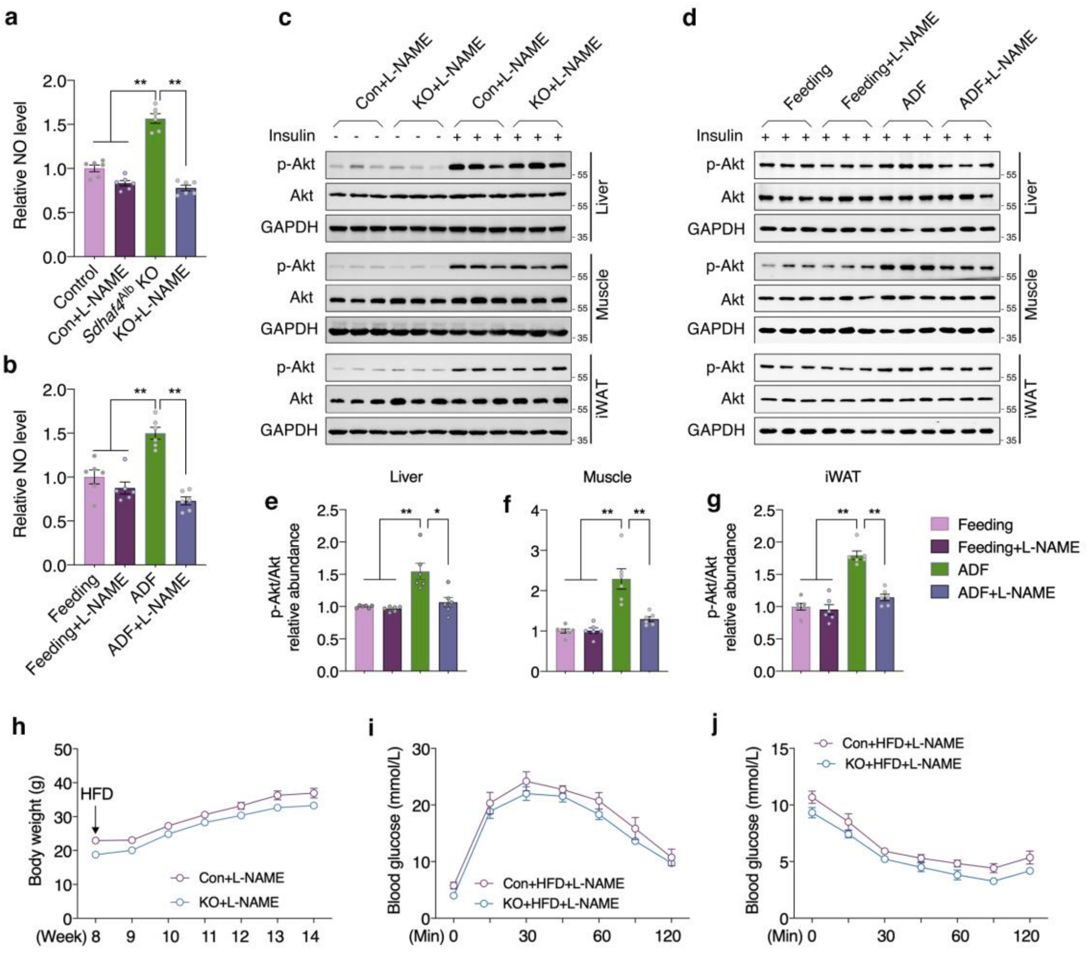

Figure S8 (Related to Figure 6). Inhibition of NO production abolishes metabolic benefits of *Sdhaf4*^Alb KO mice. a, Relative NO level of control and *Sdhaf4*^Alb KO mice under L-NAME supplement. **b,** Relative NO level of regular feeding and ADF intervened mice under L-NAME supplement. **c,** Immunoblots analysis of p-Akt level in iWAT, muscle, and liver tissues of control and *Sdhaf4*^Alb KO mice under L-NAME supplement with or without insulin challenge, n=3. **d-g**, Immunoblots analysis of p-Akt level in iWAT, muscle, and liver tissues of regular feeding and ADF intervened mice under L-NAME supplement with or without insulin challenge, n=6. **h**, Body weight curve of control and *Sdhaf4*^Alb KO mice supplement with L-NAME for 7 weeks and HFD for 6 weeks, n=8. **i-j**, Glucose tolerance test (**i**) and insulin tolerance test (**j**) for control and *Sdhaf4*^Alb KO mice supplement with L-NAME for 7 weeks and HFD for 6 weeks, n=8. Values are mean ± SEM, *p< 0.05, **p< 0.01.

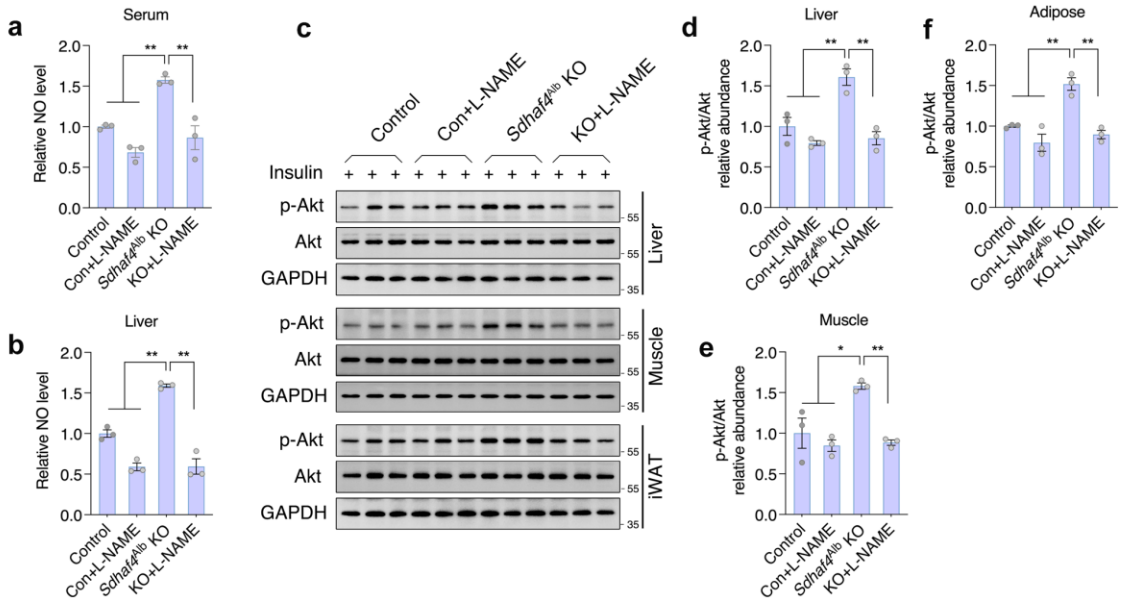

Figure S9 (Related to Figure 6). Short-term inhibition of NO production suppresses insulin signaling in target tissues. **a-b**, Serum NO (**a**) and hepatic NO (**b**) level in the control and *Sdhaf4*^{Alb} KO mice with or without L-NAME supplement for 24 h, n=3. **c-f**, Immunoblots analysis of p-Akt level in iWAT, muscle, and liver tissues of control and *Sdhaf4*^{Alb} KO mice under L-NAME supplement for 24h, followed by insulin challenge for 15 min, n=3. Values are mean ± SEM, *p< 0.05, **p< 0.01.

Supplemental Table 1

Species	Gene	Primer (5’-3’)
Mouse	*Gapdh*	Forward: CTTCAACAGCAACTCCCACTCTTCC
		Reverse: GGTGGTCCAGGGTTTCTTACTCC
Mouse	*Sdhaf1*	Forward: AAGTTCTGAGCCTGTACCGC
		Reverse: TTAAACGAGCGCGGCAAAAA
Mouse	*Sdhaf2*	Forward: ACACACAGGGCAGTCATCTG
		Reverse: ATAGCACCCTTGGGCTTGAC
Mouse	*Sdhaf3*	Forward: TAAGACTGTTGGTCCTGGCG
		Reverse: GGGAGAGAGGTGCCAAAACA
Mouse	*Sdhaf4*	Forward: AGGGACGCTGCATTGACTTT
		Reverse: GACTCTGCCCTGACACACTT
Mouse	*Asl*	Forward: CTCTCAACCTGCTGTCGCTA
		Reverse: CTGTCTCTCTTTGCATTGGCA
Mouse	*Ass1*	Forward: ACCTCCGGGTCTCTACACAA
		Reverse: ATTCAGGGCTGTGCCAGAAA
Mouse	*Nos1*	Forward: CGGGTGTCGACAATCCAAGA
		Reverse: GGGAGGATCCAGTTAGGAGC
Mouse	*Nos2*	Forward: ACAGGGAGAAAGCGCAAAAC
		Reverse: TGCTGTGCTACAGTTCCGAG
Mouse	*Nos3*	Forward: CATGGGCAACTTGAAGAGTGTG
		Reverse: TAGGTGATGCTGCCCACTTC
Mouse	*Slc7a1*	Forward: GAAGGGCTCATTGTGGATCTCT
		Reverse: CCGTCACGTAGCTGTAGAGG
Mouse	*Sdha*	Forward: AGAGATACGCACCTGTTGCC
		Reverse: ACTGGGATGGGCTCCTTAGT
Mouse	*Sdhb*	Forward: TTCCACTCGTTGGCGCTTAG
		Reverse: CCTCGACAGGCCTGAAACTG
Mouse	*Sdhc*	Forward: TGCGTTCTTGCTGAGACAT
		Reverse: AGAGAGACCCCTCCACTCAA
Mouse	*Sdhd*	Forward: CCCAGCACATTCACCTGTCA
		Reverse: GTCCCCATGAACGTAGTCGG
Mouse	*ND1*	Forward: CATTCTAATCGCCATAGCCT
		Reverse: GTTGTTAAAGGGCGTATTGG
Mouse	*ND2*	Forward: AAATCCTATCACCCTTGC
		Reverse: TTTGTTGCTGCTTCAGTT
Mouse	*ND3*	Forward: CTGACTCCCCCAAATAAATCT
		Reverse: TGAATTGCTCATGGTAGTGG
Mouse	*ND4*	Forward: CCATGTAGGAACCCTAAACC

		Reverse: TTCCTCATAGGGAGAGAAGG
Mouse	*ND4L*	Forward: GGACACTTATATTTCGCTCT
		Reverse: TTGGACGTAATCTGTTCCGTA
Mouse	*ND5*	Forward: TATAACCGCATCGGAGAC
		Reverse: TGGTAGTCATGGGTGGAG
Mouse	*Ndufs1*	Forward: CTCCTCTTGCCCTTGACTGG
		Reverse: CCAGCCCTTCATTACAGGCA
Mouse	*Ndufa1*	Forward: GAGAGGTAAAGCCGGGTCAC
		Reverse: GACCAAGCACACCCCCATAA
Mouse	*Cytb*	Forward: CTGTTCGCAGTCATAGCC
		Reverse: AAGAATCGGGTCAAGGTG
Mouse	*Cox1*	Forward: GAGCGGGAATAGTAGGCACC
		Reverse: CGGCTAGAGGTGGGTAGACT
Mouse	*Cox2*	Forward: GCCGACTAAATCAAGCAA
		Reverse: TAGGACAATGGGCATAAA
Mouse	*Cox3*	Forward: CGAAACCACATAAATCAAG
		Reverse: GTCGTAGTAGGCAAACAA
Mouse	*Cox6c*	Forward: AGCGTCTGCGGGTTCATATT
		Reverse: CGCCAAACTTATAGGCAGCG
Mouse	*Cox7c*	Forward: ATTTCTTCCGCCTTCCGTGT
		Reverse: CCGCCACTTGTTTTCCACTG
Mouse	*Uqcrq*	Forward: TGAGCCACGCGTCTATCTTC
		Reverse: TTGGCTGTAGCAGTCAAGGG
Mouse	*Uqcrb*	Forward: TCTCAGGTCAAAATGGCGGG
		Reverse: GTATGGTGAGACCAGGCACA
Mouse	*Atp6*	Forward: CCTATTCCCATCCTCAAA
		Reverse: GGGTTCATGTTCGTCCTT
Mouse	*Atp8*	Forward: ACTGGCACCTTCACCAAA
		Reverse: GGTAATGAATGAGGCAAATAGA
Mouse	*Atp5pb*	Forward: TCCAGGGGTATTACAGGCAAC
		Reverse: ATTGGCTGAGCTTGAGCCTT
Mouse	*Atp5b*	Forward: GTTGGTCCTGAGACCTTGGG
		Reverse: TCCGATTTTCCCACCCTTGG
